# Supplementary material for: Immunopeptidomics-based identification of naturally presented non-canonical circRNA-derived peptides
Source: Nat Commun. 2024 Mar 15;15:2357. doi: 10.1038/s41467-024-46408-3 (PMC10943130; doi:10.1038/s41467-024-46408-3)
Supplement: Supplementary file 1 — Supplementary information [file 41467_2024_46408_MOESM1_ESM.pdf]

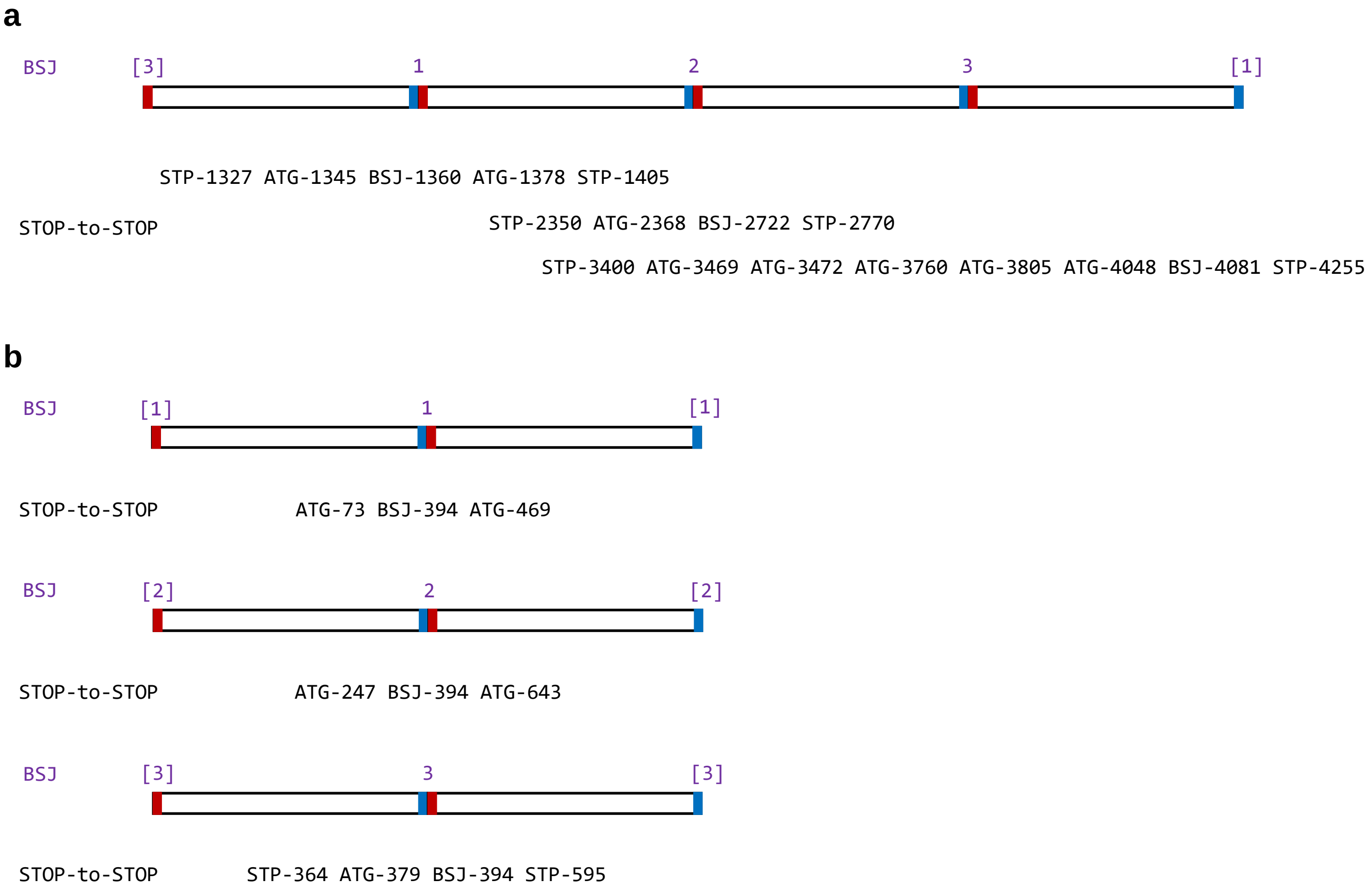

Supplementary Figure 1. Method for the in silico generation and translation of BSJ-containing fragments in circRNAs. (**a**) Four copies of a given circRNA sequence were concatenated into a single sequence ("4x circRNA") and BSJs located within flanking STOP codons. In this example, three stop-to-stop fragments contain a single BSJ translated in one of the three reading frames. (**b**) If the circRNA sequence contains an integral number of codons, only two concatenated circRNA sequences are required since translation through the BSJ does not produce a translation frame change. Open boxes represent the circRNA sequence; blue and red boxes represent the 3'- and 5'-nucleotides contributing to the BSJ; numbers above the sequence boxes indicate sequential BSJs through the concatenated circRNA construct (partial BSJs are in square brackets).

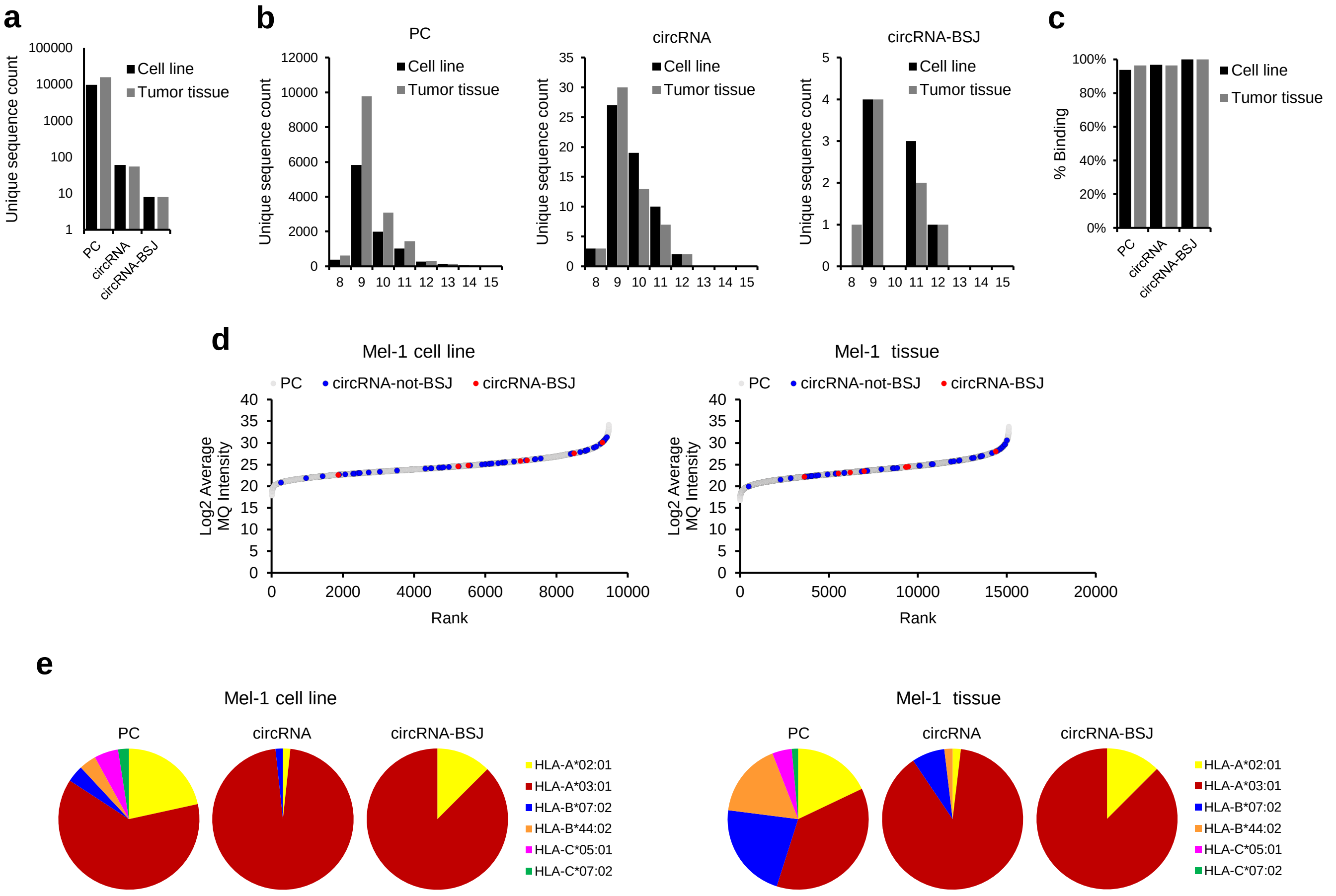

Supplementary Figure 2. Overview of the immunopeptidomics analysis in Mel-1 patient. (**a**) Total number of peptide sequences, (**b**) peptide length distribution and (**c**) percentage of peptides predicted as HLA binders (% rank < 2), in Mel-1 cell line and tumor tissues, in the PC, circRNA, and circRNA-BSJ groups. (**d**) Rank plots of PC, circRNA-not-BSJ and circRNA-BSJ derived peptide intensities in Mel-1 cell line and tumor tissues. (**e**) Predicted HLA restriction of PC, circRNA-not-BSJ and circRNA-BSJ derived peptides in cell line and tumor tissues from Mel-1 patient. Source data are provided as a Source Data file.

Gene ID	circRNAs_frame	Infinite translation candidate	Peptide Sequence	Length (AA)	NetMHCpan - 4.1 Binder/Best Allele	BSJ context (3'Exon -> Peptide [BSJ] <- 5'Exon)	Patient ID	Detection
ANXA6	hsa_circ_0074614_0	TRUE	VQDLIADLK	9	no_binder	VVLLQGTREEDDVVSEDLVQQD->V**[QD]**LIADLK<-YELTGKFERLIVGLMRP	T1185B	Cell line

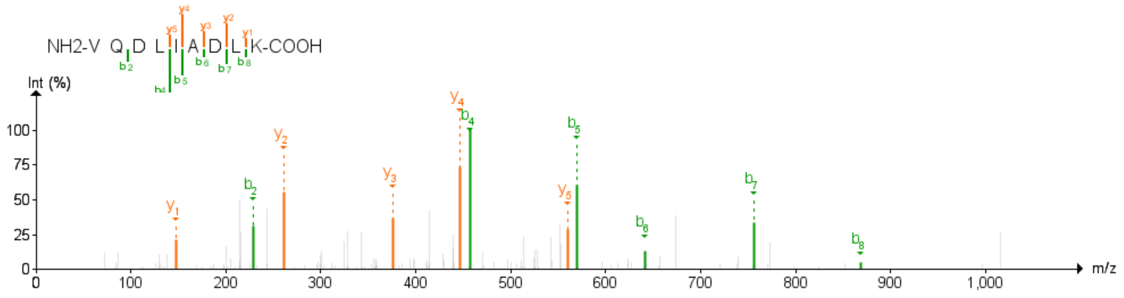

Gene ID	circRNAs_frame	Infinite translation candidate	Peptide Sequence	Length (AA)	NetMHCpan - 4.1 Binder/Best Allele	BSJ context (3'Exon -> Peptide [BSJ] <- 5'Exon)	Patient ID	Detection
CBLL1	hsa_circ_0081903_0	FALSE	DTFFGTFR	8	HLA-A*68:01	GMTVKGVSCLQISEDFL->DTFFGTF**[R]**<-RI	T1185B	Cell line

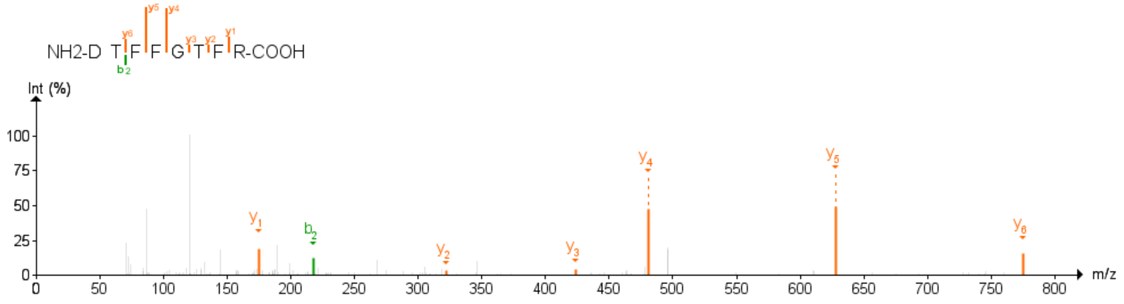

Gene ID	circRNAs_frame	Infinite translation candidate	Peptide Sequence	Length (AA)	NetMHCpan - 4.1 Binder/Best Allele	BSJ context (3'Exon -> Peptide [BSJ] <- 5'Exon)	Patient ID	Detection
CDC73	hsa_circ_0111569_0	TRUE	TTENIPVVRR	10	HLA-A*68:01	KEETEGFKIDTMGTYHGMTLKSV->**[TT]**ENIPVVRR<-PDRKDLLGYLNGEAS	T1185B	Cell line

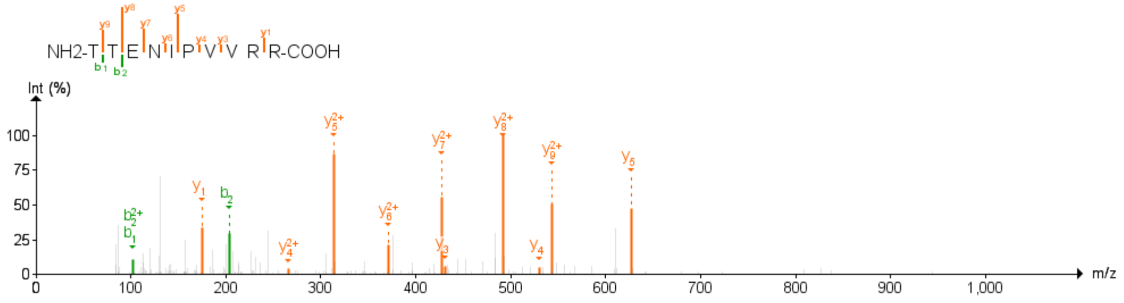

Gene ID	circRNAs_frame	Infinite translation candidate	Peptide Sequence	Length (AA)	NetMHCpan - 4.1 Binder/Best Allele	BSJ context (3'Exon -> Peptide [BSJ] <- 5'Exon)	Patient ID	Detection
CLASP2	hsa_circ_0123550_2	FALSE	LETLGDKECI	10	no_binder	SFSVWDEHFKTILLLL->LETLGDK**[EC]**I<-	T1185B	Cell line

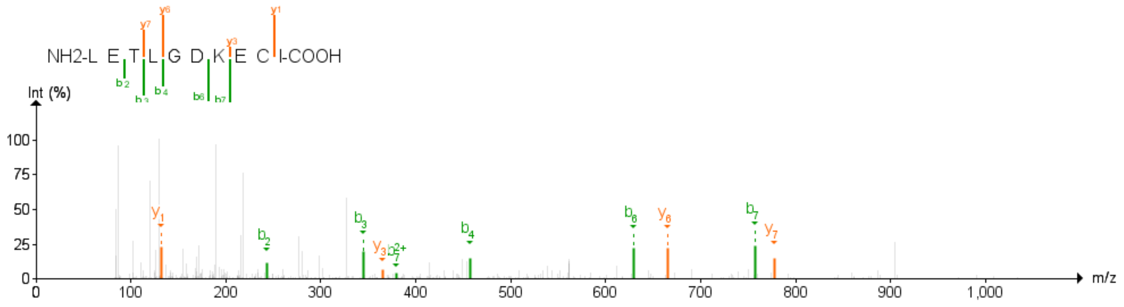

Gene ID	circRNAs_frame	Infinite translation candidate	Peptide Sequence	Length (AA)	NetMHCpan - 4.1 Binder/Best Allele	BSJ context (3'Exon -> Peptide [BSJ] <- 5'Exon)	Patient ID	Detection
CTNNB1	hsa_circ_0003137_1	FALSE	RVFEVYHTTVLK	12	HLA-A*68:01	MATKKA->[R]VFEVYHTTVLK<-IQRGQWLLKLI	T1185B	Cell line
	hsa_circ_0004030_1	FALSE				SWMGCLQVTAISWPGLILTCKSSF->[R]VFEVYHTTVLK<-IQRGQWLLKLI		

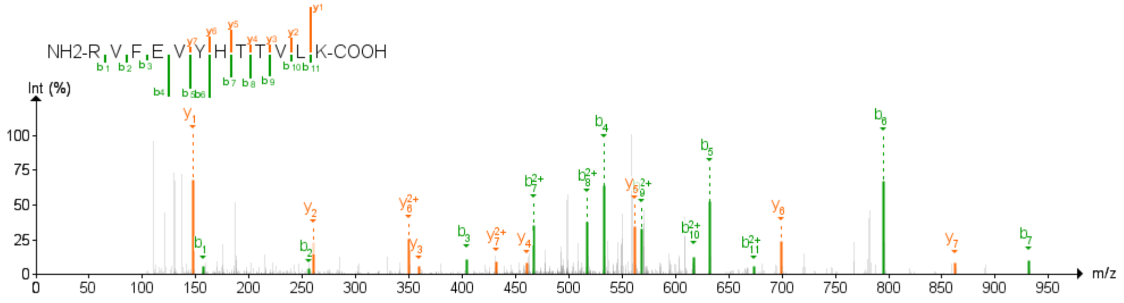

Gene ID	circRNAs_frame	Infinite translation candidate	Peptide Sequence	Length (AA)	NetMHCpan - 4.1 Binder/Best Allele	BSJ context (3'Exon -> Peptide [BSJ] <- 5'Exon)	Patient ID	Detection
CTSB	hsa_circ_0083357_1	FALSE	STMSTNGIPRGR	12	HLA-A*68:01	GAGPLSIPCRMSW->STMSTNGIPRG[R]<-GRGREGT	T1185B	Cell line
			STNGIPRGR	9	HLA-A*68:01	GAGPLSIPCRMSWSTM->STNGIPRG[R]<-GRGREGT		

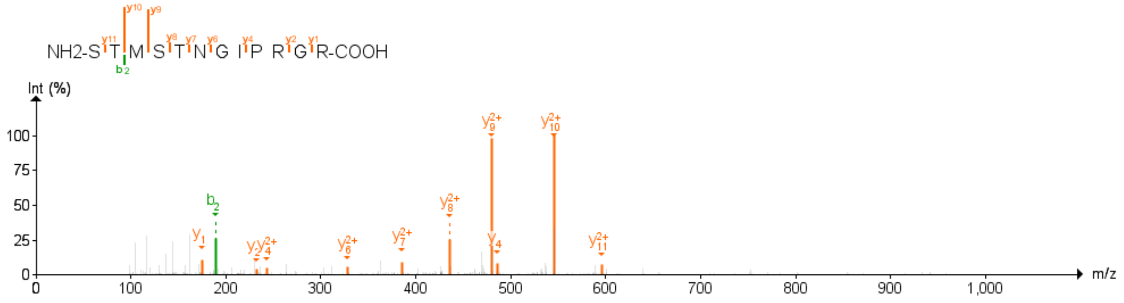

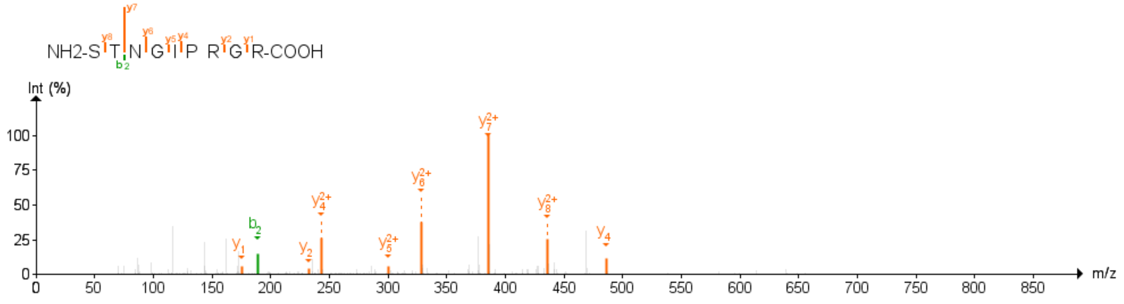

Gene ID	circRNAs_frame	Infinite translation candidate	Peptide Sequence	Length (AA)	NetMHCpan - 4.1 Binder/Best Allele	BSJ context (3'Exon -> Peptide [BSJ] <- 5'Exon)	Patient ID	Detection
ETV5	hsa_circ_0068440_2	TRUE	FVPDFQSDNR	10	HLA-A*68:01	QEAWLAEAQVPDDEQ->FVPDFQSDN[R]<-GSLFPQKLLNAETSQSGIRDAEST	T1185B	Cell line

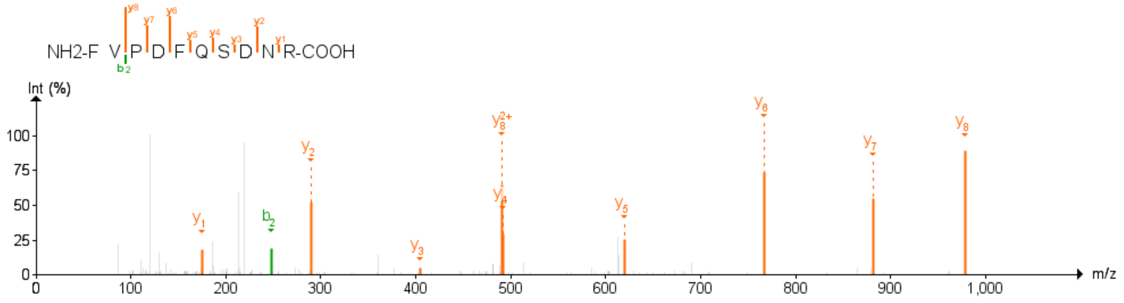

Gene ID	circRNAs_frame	Infinite translation candidate	Peptide Sequence	Length (AA)	NetMHCpan - 4.1 Binder/Best Allele	BSJ context (3'Exon -> Peptide [BSJ] <- 5'Exon)	Patient ID	Detection
GAS7	hsa_circ_0042080_0	FALSE	EAWNGPPSAGR	11	HLA-A*68:01	WERPSSSPGIPASPGSHRSSLPPT->[E]AWNGPPSAGR<-RKPDGHPSTWLAEL	T1185B	Cell line

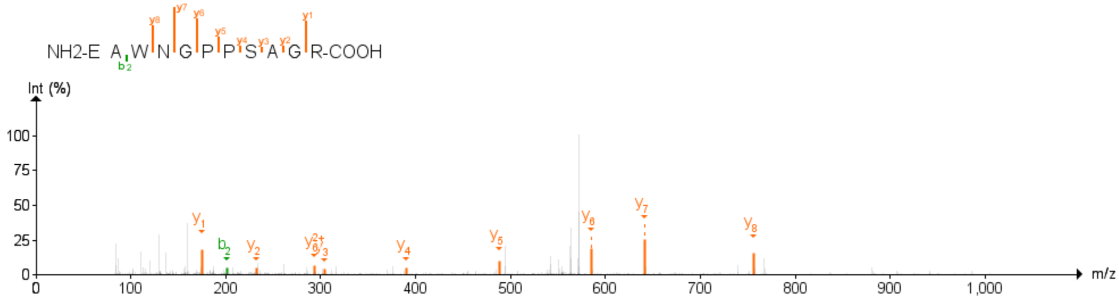

Gene ID	circRNAs_frame	Infinite translation candidate	Peptide Sequence	Length (AA)	NetMHCpan - 4.1 Binder/Best Allele	BSJ context (3'Exon -> Peptide [BSJ] <- 5'Exon)	Patient ID	Detection
KIAA0226	hsa_circ_0068719_1	FALSE	SSITGLSVTR	10	HLA-A*68:01	MVAWSGFAGTCRA->SSITGLSVT[R]<-REHWQLLGNLKTTVEGLVSTNSPN	T1185B	Cell line

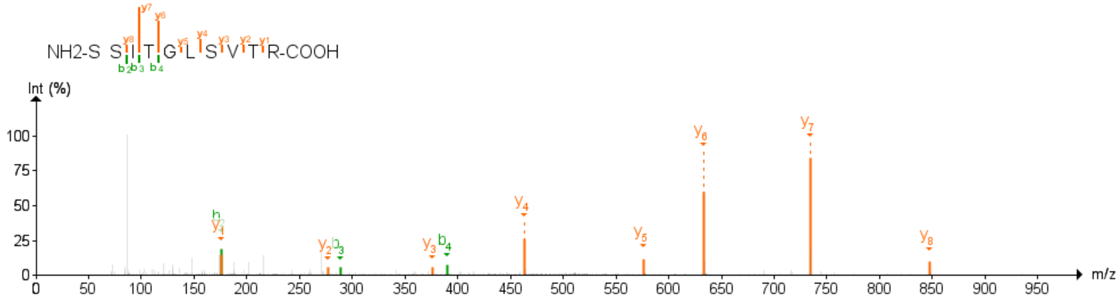

Gene ID	circRNAs_frame	Infinite translation candidate	Peptide Sequence	Length (AA)	NetMHCpan - 4.1 Binder/Best Allele	BSJ context (3'Exon -> Peptide [BSJ] <- 5'Exon)	Patient ID	Detection
LIN9	hsa_circ_0016709_0	FALSE	EIYSNNVNTR	10	HLA-A*68:01	ADVSQFKDLPDEIPLPLVIGTKVT->[E]IYSNNVNTR<-	T1185B	Cell line

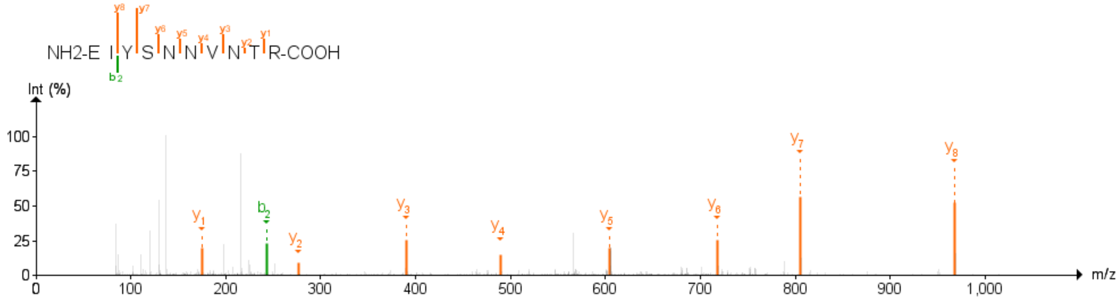

Gene ID	circRNAs_frame	Infinite translation candidate	Peptide Sequence	Length (AA)	NetMHCpan - 4.1 Binder/Best Allele	BSJ context (3'Exon -> Peptide [BSJ] <- 5'Exon)	Patient ID	Detection
LINC00221	hsa_circ_0101415_1	FALSE	AAAAFRFPR	9	HLA-A*68:01	MPARSWLGE->AAAAFRFP**[R]**<-DFCVCAHIDVPTLCPSQ	T1185B	Cell line
			EAAAAFRFPR	10	HLA-A*68:01	MPARSWLG->EAAAAFRFP**[R]**<-DFCVCAHIDVPTLCPSQ		
	hsa_circ_0101416_0	FALSE	AAAAFRFPR	9	HLA-A*68:01	MPARSWLGE->AAAAFRFP**[R]**<-M		
			EAAAAFRFPR	10	HLA-A*68:01	MPARSWLG->EAAAAFRFP**[R]**<-M		

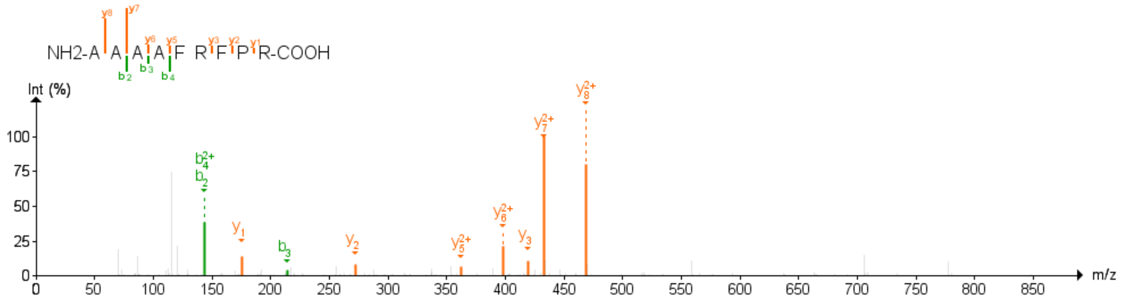

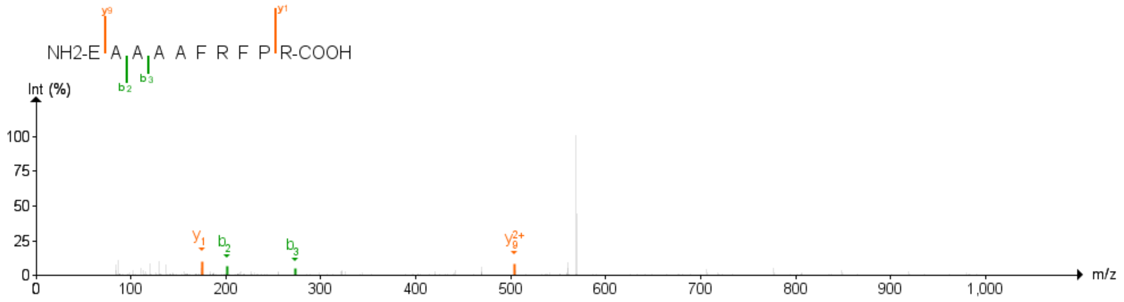

Gene ID	circRNAs_frame	Infinite translation candidate	Peptide Sequence	Length (AA)	NetMHCpan - 4.1 Binder/Best Allele	BSJ context (3'Exon -> Peptide [BSJ] <- 5'Exon)	Patient ID	Detection
NOD1	hsa_circ_0079703_1	FALSE	VPAPPSLTR	9	HLA-A*68:01	MTTSRPKMRRLC->VPAPPSLT**[R]**<-GCAE	T1185B	Cell line

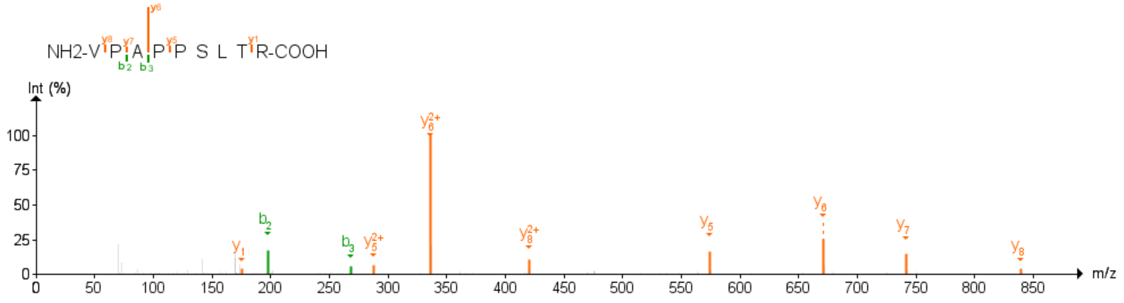

Gene ID	circRNAs_frame	Infinite translation candidate	Peptide Sequence	Length (AA)	NetMHCpan - 4.1 Binder/Best Allele	BSJ context (3'Exon -> Peptide [BSJ] <- 5'Exon)	Patient ID	Detection
PAFAH1B2	hsa_circ_0095083_1	FALSE	STTDLFWTVK	10	HLA-A*68:01	MENWRILSL->**[S]**TTDLFWTVK<-TKSLMYCSWETPWCS	T1185B	Cell line

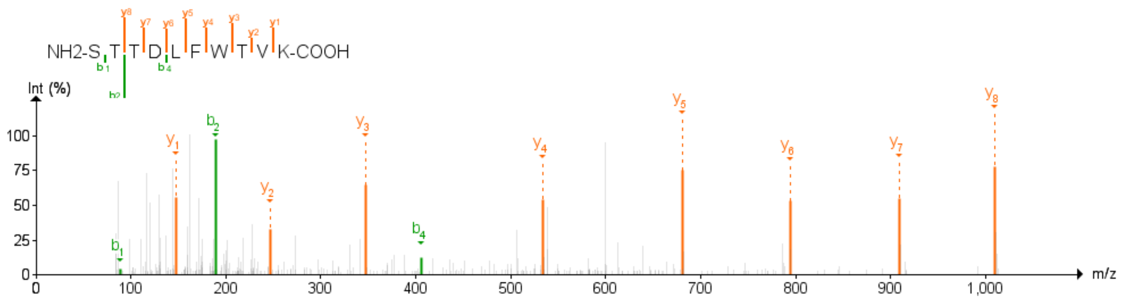

Gene ID	circRNAs_frame	Infinite translation candidate	Peptide Sequence	Length (AA)	NetMHCpan - 4.1 Binder/Best Allele	BSJ context (3'Exon -> Peptide [BSJ] <- 5'Exon)	Patient ID	Detection
PKNOX1	hsa_circ_0061852_2	FALSE	QTPFAFHPR	9	HLA-A*68:01	VVTPQGQVVTQTLSPGTIRIQNS->[QT]PFAFHPR<-	T1185B	Cell line
	hsa_circ_0116109_2	FALSE				KTKMNSETLLSGEPGSPYSPVQS->[QT]PFAFHPR<-		
	hsa_circ_0116112_2	FALSE				TPVNMNVDSLQSLSSDGATLAVQ->[QT]PFAFHPR<-		

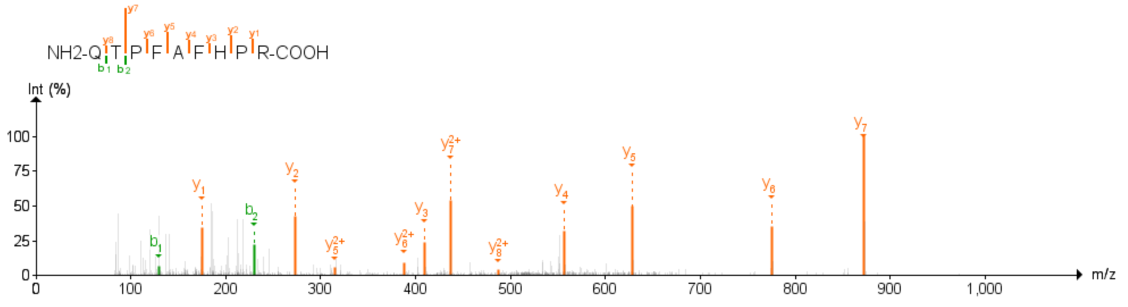

Gene ID	circRNAs_frame	Infinite translation candidate	Peptide Sequence	Length (AA)	NetMHCpan - 4.1 Binder/Best Allele	BSJ context (3'Exon -> Peptide [BSJ] <- 5'Exon)	Patient ID	Detection
PTPRG	hsa_circ_0066402_0	FALSE	NTASMAGGFLLR	12	HLA-A*68:01	MAQRAL->NTASMAGGFLL[R]<-HVTSCMEAWPVSSPALFVPSQPAP	T1185B	Cell line
	hsa_circ_0066406_0	FALSE				MAQRAL->NTASMAGGFLL[R]<-CLWS		
	hsa_circ_0124401_0	FALSE				MAQRAL->NTASMAGGFLL[R]<-YYRTCSD		

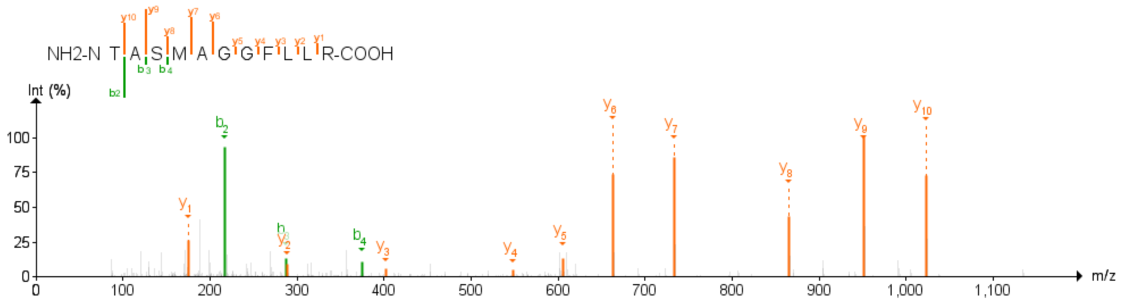

Gene ID	circRNAs_frame	Infinite translation candidate	Peptide Sequence	Length (AA)	NetMHCpan - 4.1 Binder/Best Allele	BSJ context (3'Exon -> Peptide [BSJ] <- 5'Exon)	Patient ID	Detection
RFWD2/ COP1	hsa_circ_0015364_0	FALSE	DLYNGSSIVSR	11	HLA-A*68:01	YNSVRPLATLSYAS->DLYNGSSIVS[R]<-QRNSLGIIAR	T1185B	Cell line
	hsa_circ_0015366_0	FALSE				YNSVRPLATLSYAS->DLYNGSSIVS[R]<-NHMQPNYRFLWNSSRLQEEIRESN		
	hsa_circ_0111261_0	FALSE				YNSVRPLATLSYAS->DLYNGSSIVS[R]<-K		
	hsa_circ_0111262_1	FALSE				YNSVRPLATLSYAS->DLYNGSSIVS[R]<-TSGTMMNRSGDSGAS		

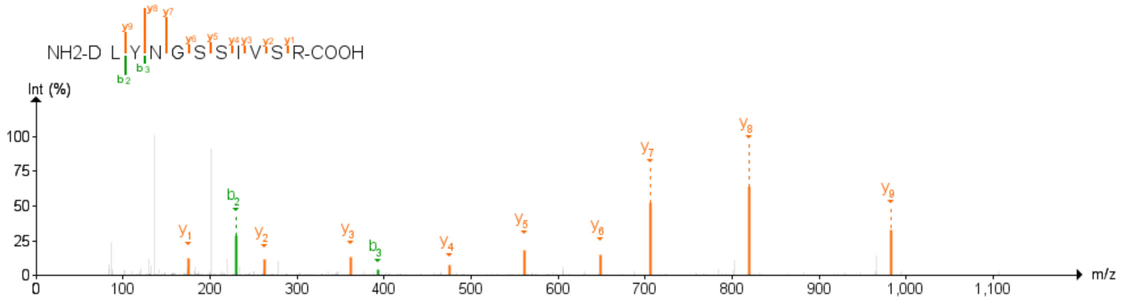

Gene ID	circRNAs_frame	Infinite translation candidate	Peptide Sequence	Length (AA)	NetMHCpan - 4.1 Binder/Best Allele	BSJ context (3'Exon -> Peptide [BSJ] <- 5'Exon)	Patient ID	Detection
STAT3	hsa_circ_0043818_1	TRUE	TIYEESSSFFR	11	HLA-A*68:01	SIAASCKSRMFSIS->TIYEESSSFF[R]<-VSGAAAAQTGRGSRGFRRRSRGNK	T1185B	Cell line
	hsa_circ_0106859_1	FALSE				SIAASCKSRMFSIS->TIYEESSSFF[R]<-MAQWNQLQQLDTRYLEQLHQLYSD		

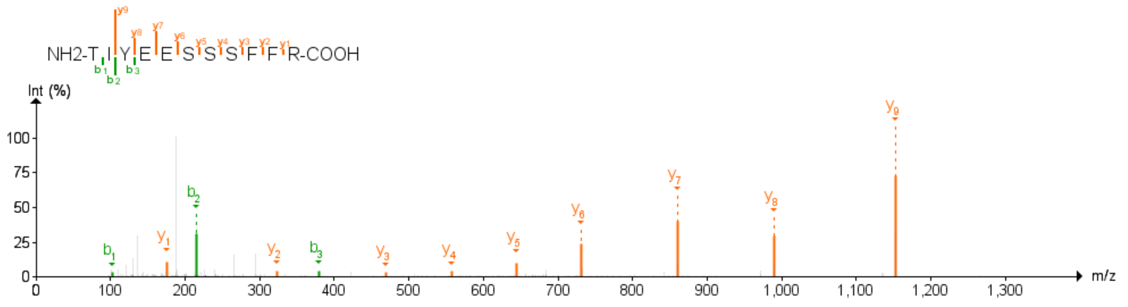

Gene ID	circRNAs_frame	Infinite translation candidate	Peptide Sequence	Length (AA)	NetMHCpan - 4.1 Binder/Best Allele	BSJ context (3'Exon -> Peptide **[BSJ]** <- 5'Exon)	Patient ID	Detection
ABCA2	hsa_circ_0089621_2	FALSE	RLKEPSTQR	9	HLA-A*03:01	ISWVYSVAMTIQHIVAEKEH->RLK**[EP]**STQR<-RP	Mel-1	Tumor

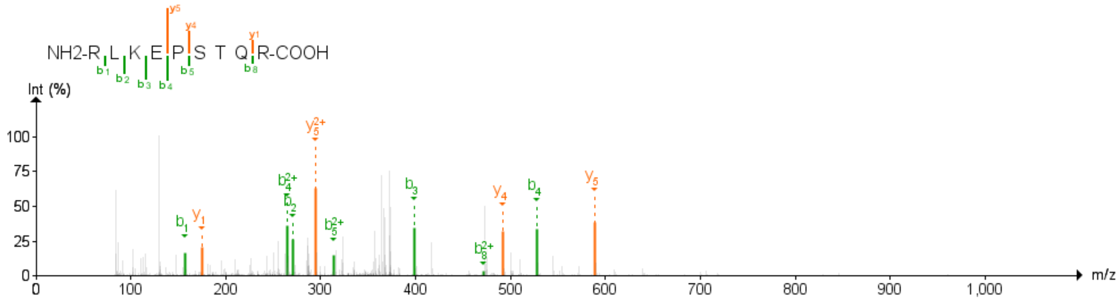

Gene ID	circRNAs_frame	Infinite translation candidate	Peptide Sequence	Length (AA)	NetMHCpan - 4.1 Binder/Best Allele	BSJ context (3'Exon -> Peptide **[BSJ]** <- 5'Exon)	Patient ID	Detection
CCAR1	hsa_circ_0018553_1	FALSE	VLSKGKPPK	9	HLA-A*03:01	ISAASITPLLQTQPQPLLQQPQQK->**[V]**LSKGKPPK<-	Mel-1	Cell line

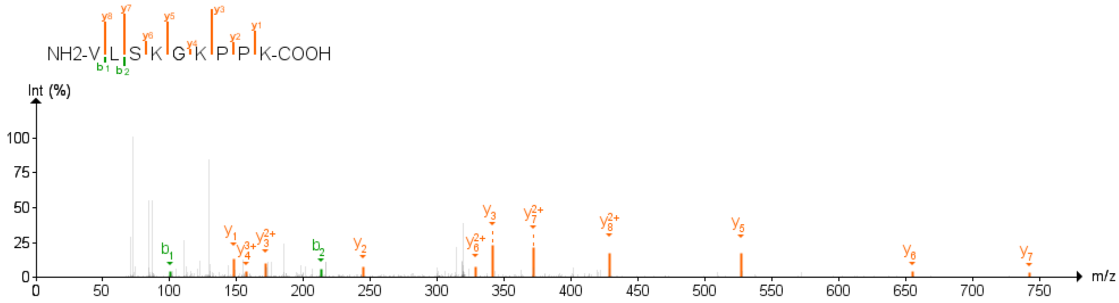

Gene ID	circRNAs_frame	Infinite translation candidate	Peptide Sequence	Length (AA)	NetMHCpan - 4.1 Binder/Best Allele	BSJ context (3'Exon -> Peptide **[BSJ]** <- 5'Exon)	Patient ID	Detection
CEP57L1	hsa_circ_0130265_1	FALSE	KLRDPTDSTLR	11	HLA-A*03:01	MGLSSCKN->KLRDPTDSTL**[R]**<-AWYLVQT	Mel-1	Cell line

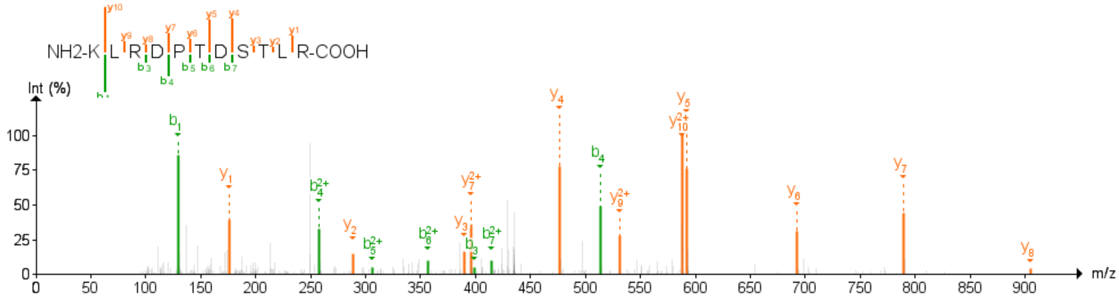

Gene ID	circRNAs_frame	Infinite translation candidate	Peptide Sequence	Length (AA)	NetMHCpan - 4.1 Binder/Best Allele	BSJ context (3'Exon -> Peptide **[BSJ]** <- 5'Exon)	Patient ID	Detection
CTNNB1	hsa_circ_0003137_1	FALSE	RVFEVYHTTVLK	12	HLA-A*03:01	MATKKA->**[R]**VFEVYHTTVLK<-IQRGQWLLKLI	Mel-1	Cell line + Tumor
	hsa_circ_0004030_1	FALSE				SWMGCLQVTAISWPGLILTCKSSF->**[R]**VFEVYHTTVLK<-IQRGQWLLKLI		

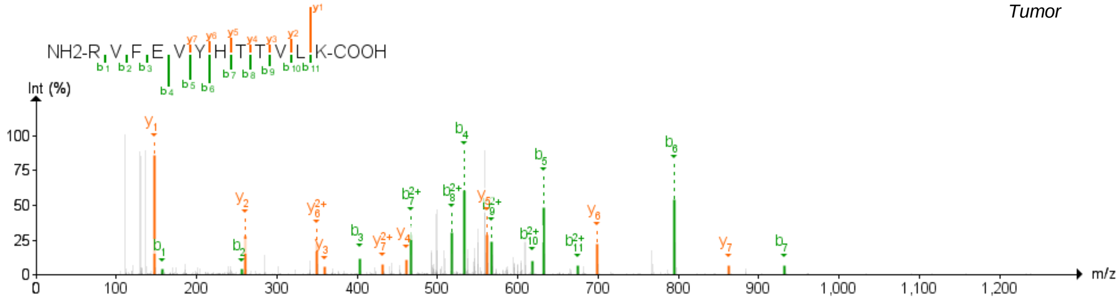

Gene ID	circRNAs_frame	Infinite translation candidate	Peptide Sequence	Length (AA)	NetMHCpan - 4.1 Binder/Best Allele	BSJ context (3'Exon -> Peptide [BSJ] <- 5'Exon)	Patient ID	Detection
MALAT1	hsa_circ_0096124_2	FALSE	KLLHGVKNVFK	11	HLA-A*03:01	M->**[KL]**LHGVKNVFK<-RKLRERTTEPRINT	Mel-1	Cell line + Tumor

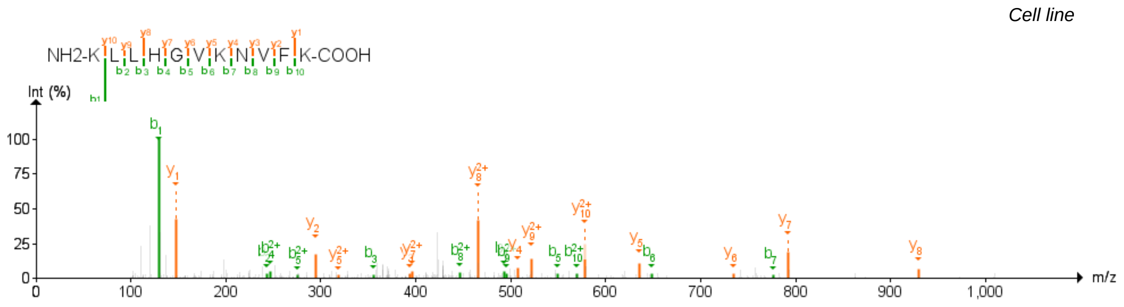

Gene ID	circRNAs_frame	Infinite translation candidate	Peptide Sequence	Length (AA)	NetMHCpan - 4.1 Binder/Best Allele	BSJ context (3'Exon -> Peptide [BSJ] <- 5'Exon)	Patient ID	Detection
PDIA3	hsa_circ_0035034_2	FALSE	RVSSYFVLH	9	HLA-A*03:01	LLERFLLLLSELLKERSLSCRRSS->**[R]**VSSYFVLH<-ISLTSLRTRLWHIQSK	Mel-1	Cell line

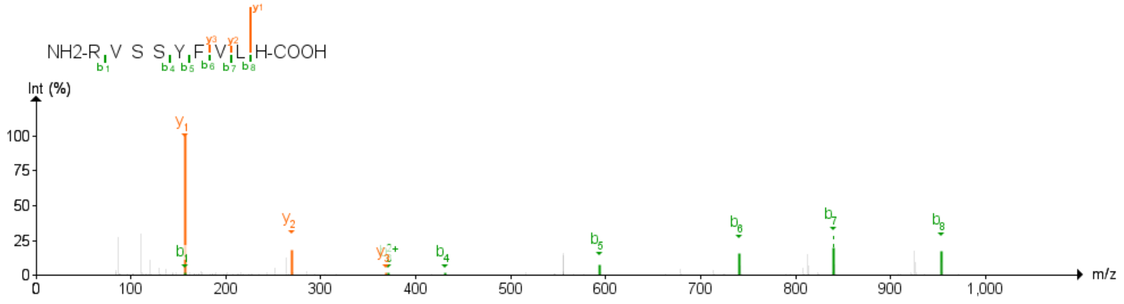

Gene ID	circRNAs_frame	Infinite translation candidate	Peptide Sequence	Length (AA)	NetMHCpan - 4.1 Binder/Best Allele	BSJ context (3'Exon -> Peptide [BSJ] <- 5'Exon)	Patient ID	Detection
SAE1	hsa_circ_0051662_2	FALSE	ILAQEIVKV	9	HLA-A*02:01	MAPVCAVVGG->ILAQEIV**[KV]**<-LLLRDGPSVCGGWRDFGTGNCEG	Mel-1	Cell line + Tumor

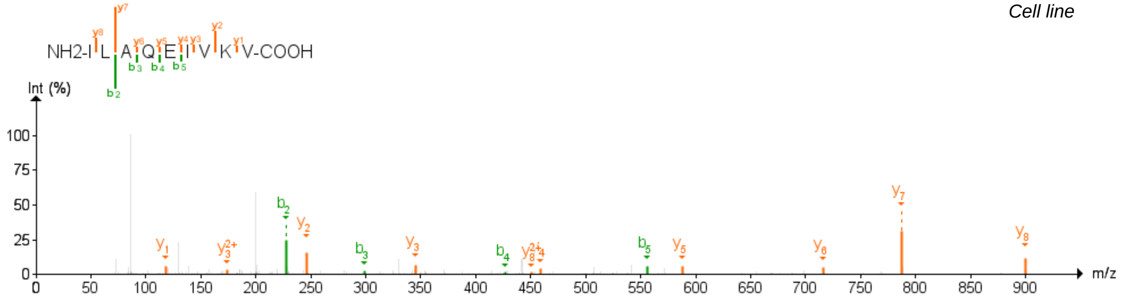

Gene ID	circRNAs_frame	Infinite translation candidate	Peptide Sequence	Length (AA)	NetMHCpan - 4.1 Binder/Best Allele	BSJ context (3'Exon -> Peptide [BSJ] <- 5'Exon)	Patient ID	Detection
PPP2R3A	hsa_circ_0122039_2	FALSE	KVSLSFTEK	9	HLA-A*03:01	SPVGDKAKDTTSAVLIQQTPEVI->**[KV]**SLSFTEK<-	Mel-1	Cell line + Tumor

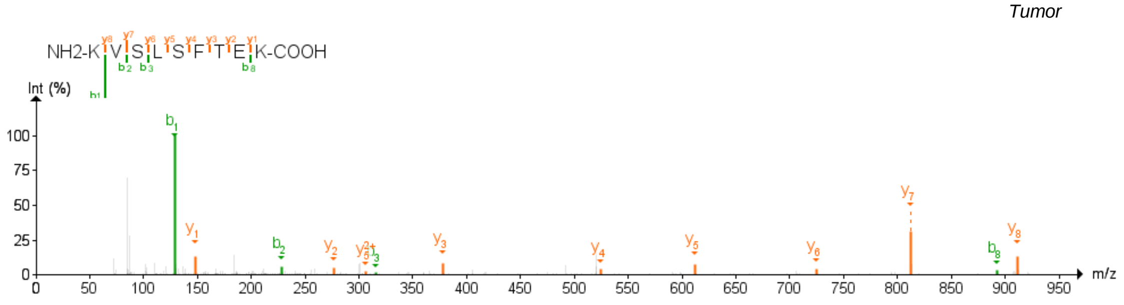

Gene ID	circRNAs_frame	Infinite translation candidate	Peptide Sequence	Length (AA)	NetMHCpan - 4.1 Binder/Best Allele	BSJ context (3'Exon -> Peptide [BSJ] <- 5'Exon)	Patient ID	Detection
TOPBP1	hsa_circ_0121989_1	FALSE	KSLAAELLVLK	11	HLA-A*03:01	MYTPHCART->K[S]LAAELLVLK<-	Mel-1	Cell line + Tumor

Tumor

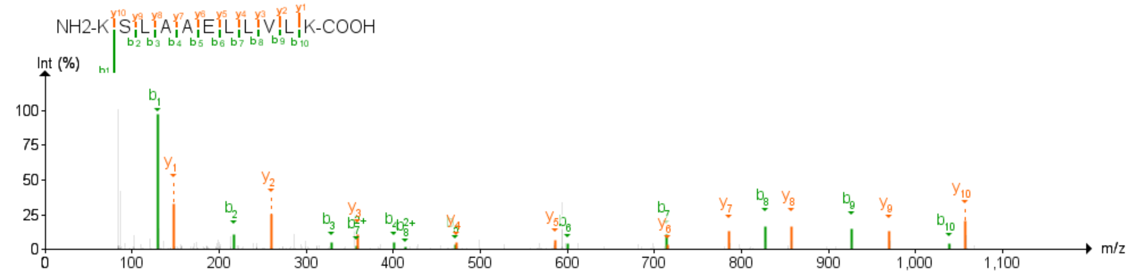

Gene ID	circRNAs_frame	Infinite translation candidate	Peptide Sequence	Length (AA)	NetMHCpan - 4.1 Binder/Best Allele	BSJ context (3'Exon -> Peptide [BSJ] <- 5'Exon)	Patient ID	Detection
WARS	hsa_circ_0033185_2	FALSE	VLTRLLNK	8	HLA-A*03:01	MPRTSSPVALTSTRLSYSLTWTTW->[V]LTRLLNK<-SSDLRPAVNVVPER	Mel-1	Tumor
	hsa_circ_0033189_1	FALSE				SDGESAALESARPAPLLILLPVCM->[V]LTRLLNK<-SSDLRPAVNVVPER		

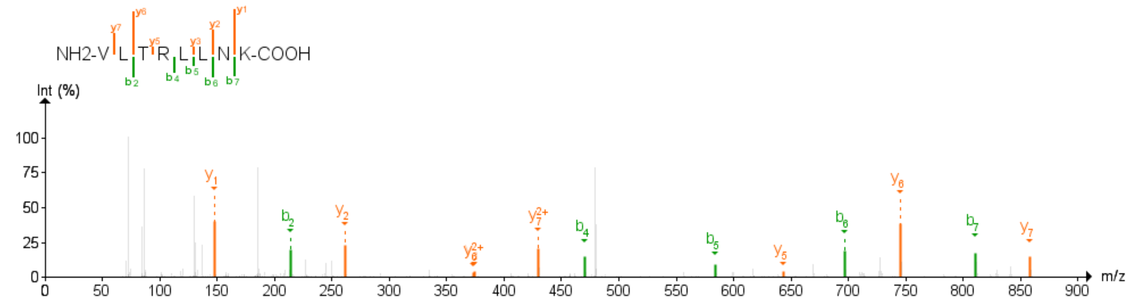

Gene ID	circRNAs_frame	Infinite translation candidate	Peptide Sequence	Length (AA)	NetMHCpan - 4.1 Binder/Best Allele	BSJ context (3'Exon -> Peptide [BSJ] <- 5'Exon)	Patient ID	Detection
ZMYM4	hsa_circ_0113154_2	FALSE	RVVVSWIQK	9	HLA-A*03:01	DKAANQVEETLHTHLPQTPETNF->[RV]VVSWIQK<-CLKI	Mel-1	Tumor

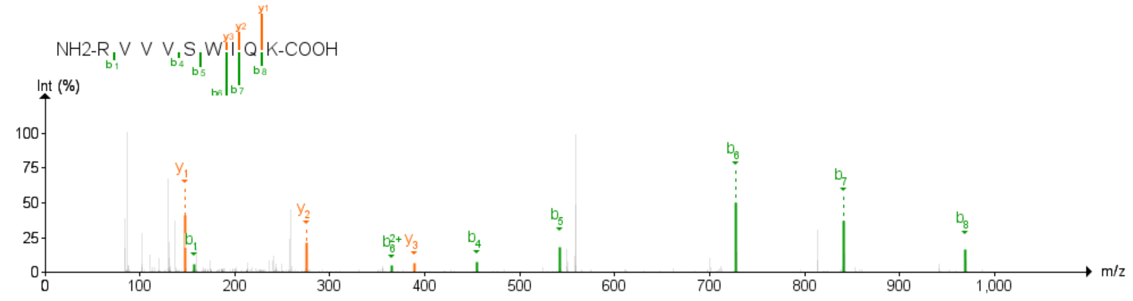

Supplementary Figure 3. Spectrum visualization of the MS identified circRNA-BSJ derived peptides. MS/MS spectra of peptides identified in T1185B and Mel-1, encoded in circRNAs from different host genes. Sample source is indicated for spectrums from peptides detected in both cell line and tissue from Mel-1 patient.

a

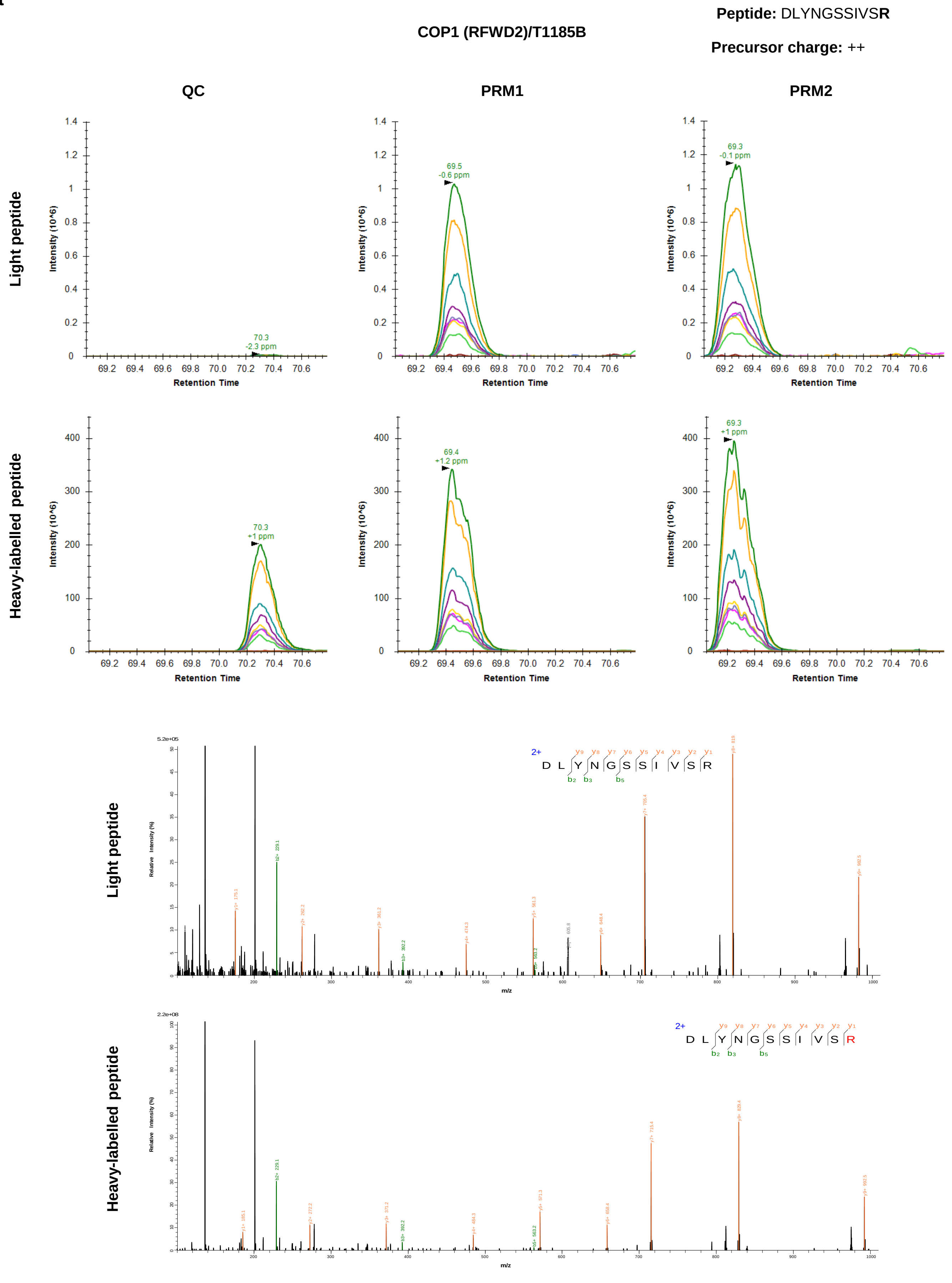
COP1 (RFWD2)/T1185B
Peptide: DLYNGSSIVSR
Precursor charge: ++
QC
PRM1
PRM2
Light peptide
Heavy-labelled peptide
Intensity (10^6)
Retention Time
70.3
-2.3 ppm
69.5
-0.6 ppm
69.3
-0.1 ppm
70.3
+1 ppm
69.4
+1.2 ppm
69.3
+1 ppm
5.2e+05
2.2e+08
Relative Intensity (%)
m/z
2+
D L Y N G S S I V S R
y9 y8 y7 y6 y5 y4 y3 y2 y1
b2 b3 b5

b

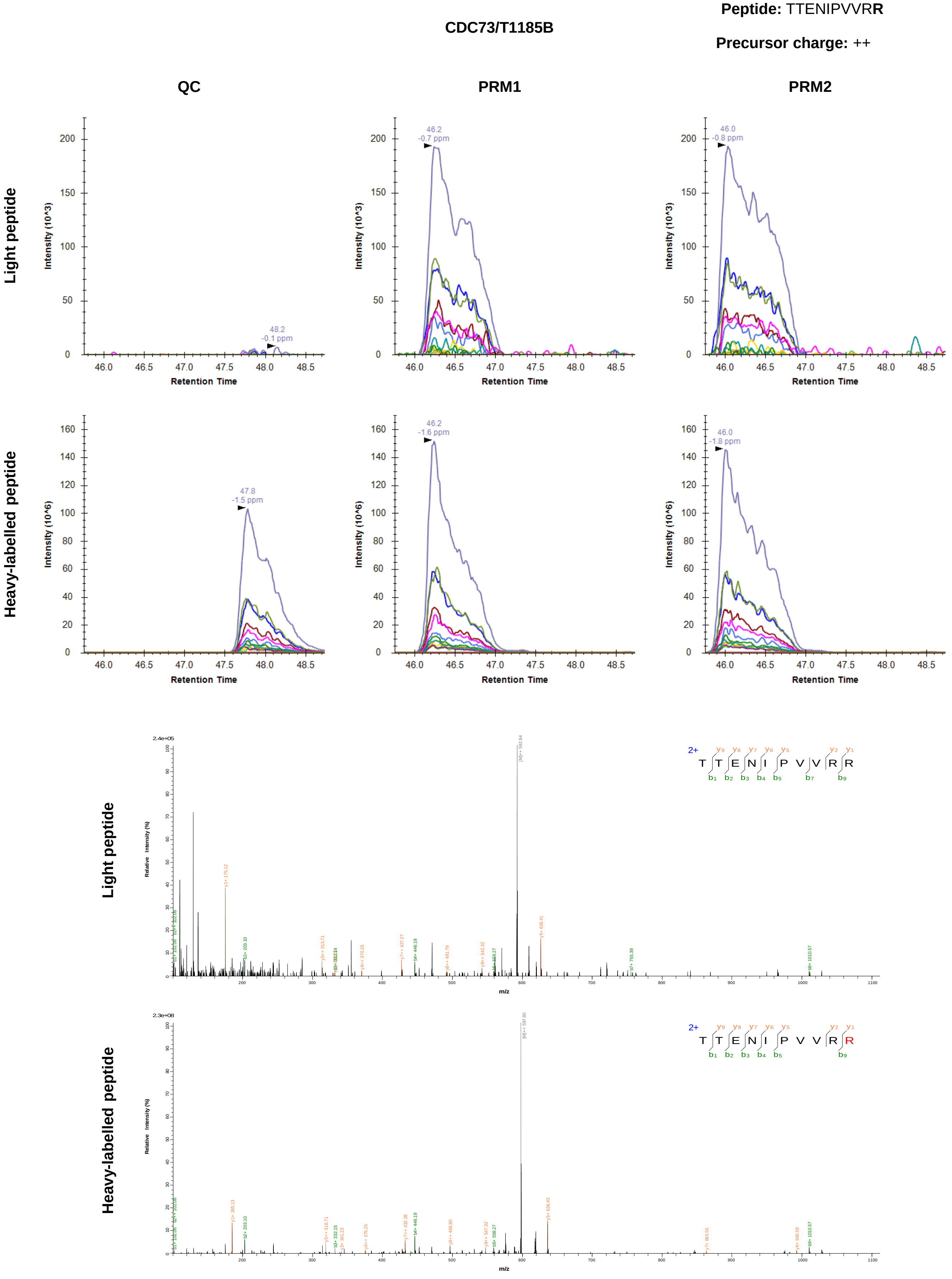

CDC73/T1185B
Peptide: TTENIPVVRR
Precursor charge: ++
QC
PRM1
PRM2
Light peptide
Heavy-labelled peptide
Intensity (10^3)
Intensity (10^6)
Retention Time
48.2 -0.1 ppm
46.2 -0.7 ppm
46.0 -0.8 ppm
47.8 -1.5 ppm
46.2 -1.6 ppm
46.0 -1.8 ppm
Light peptide
Heavy-labelled peptide
m/z
Relative Intensity (%)
2.4e+05
2.3e+08

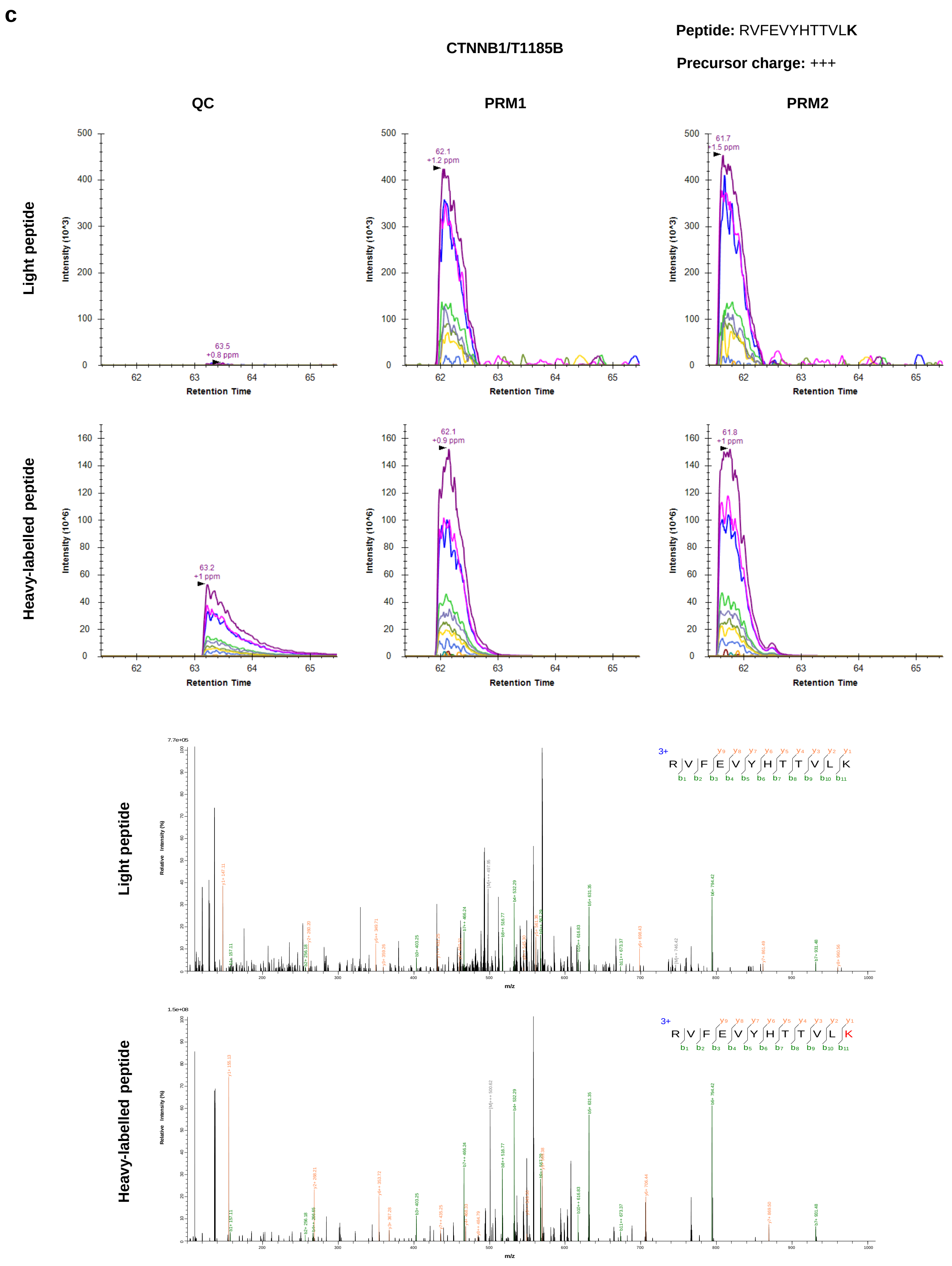
c
CTNNB1/T1185B
Peptide: RVFEVYHTTVLK
Precursor charge: +++
QC
PRM1
PRM2
Light peptide
Heavy-labelled peptide
Intensity (10^3)
Intensity (10^6)
Retention Time
63.5
+0.8 ppm
62.1
+1.2 ppm
61.7
+1.5 ppm
63.2
+1 ppm
62.1
+0.9 ppm
61.8
+1 ppm
7.7e+05
1.5e+08
Relative Intensity (%)
m/z
3+
R V F E V Y H T T V L K

d

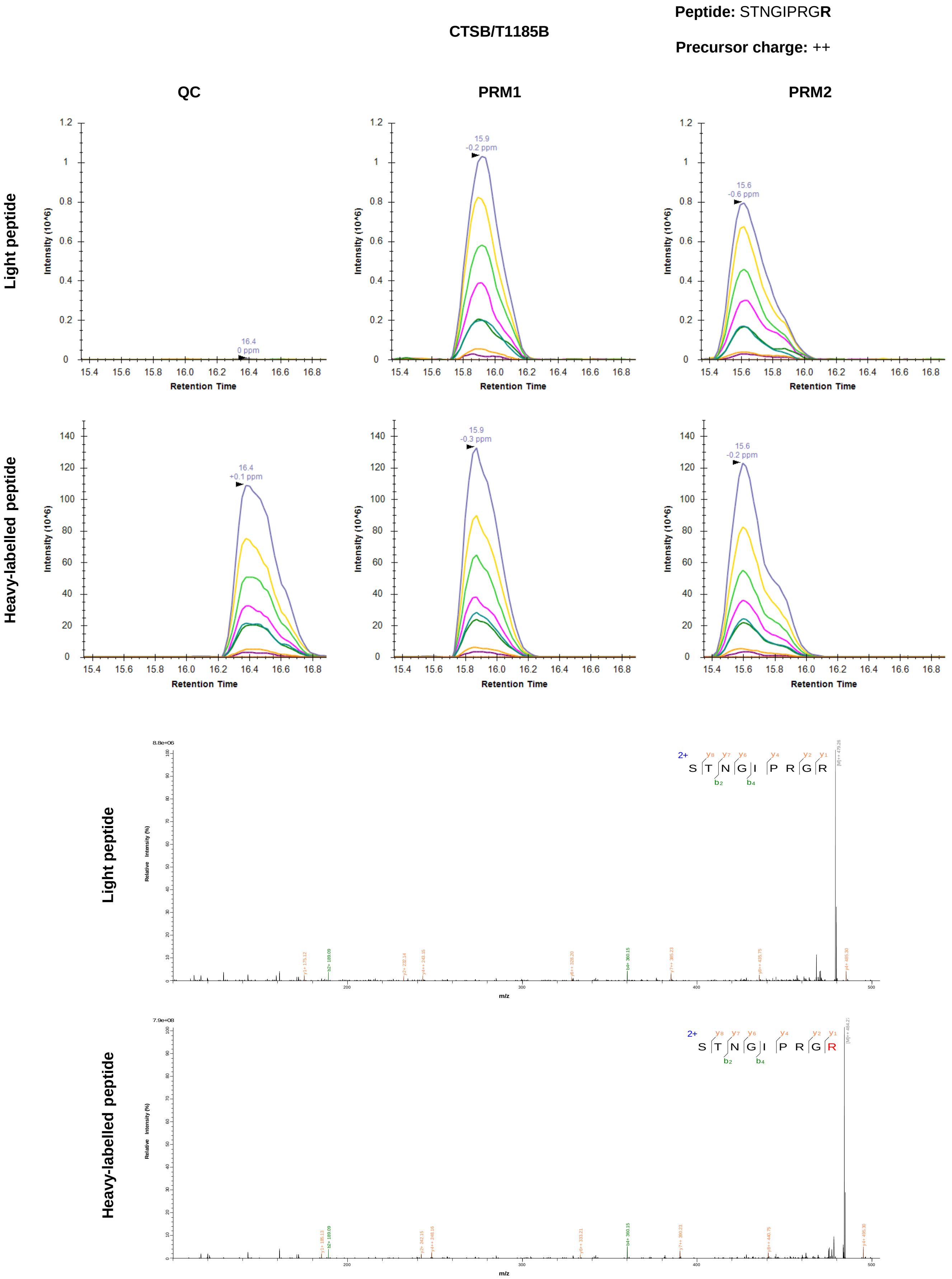

CTSB/T1185B
Peptide: STNGIPRGR
Precursor charge: ++
QC
PRM1
PRM2
Light peptide
Heavy-labelled peptide
Intensity (10^6)
Retention Time
16.4
0 ppm
15.9
-0.2 ppm
15.6
-0.6 ppm
16.4
+0.1 ppm
15.9
-0.3 ppm
15.6
-0.2 ppm
Relative Intensity (%)
m/z
8.8e+06
7.9e+08

e

GAS7/T1185B

Peptide: EAWNGPPSAG**R**

Precursor charge: ++

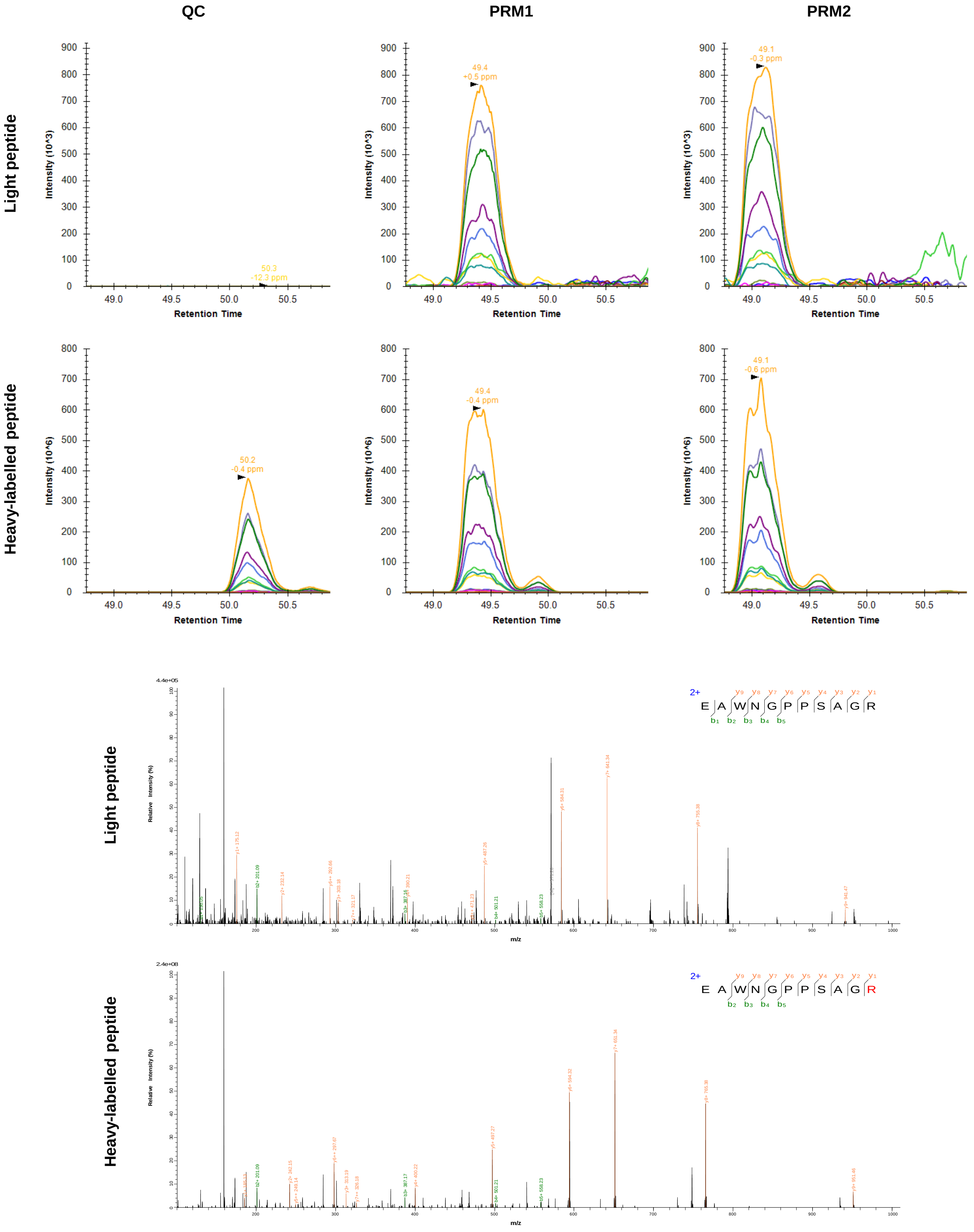

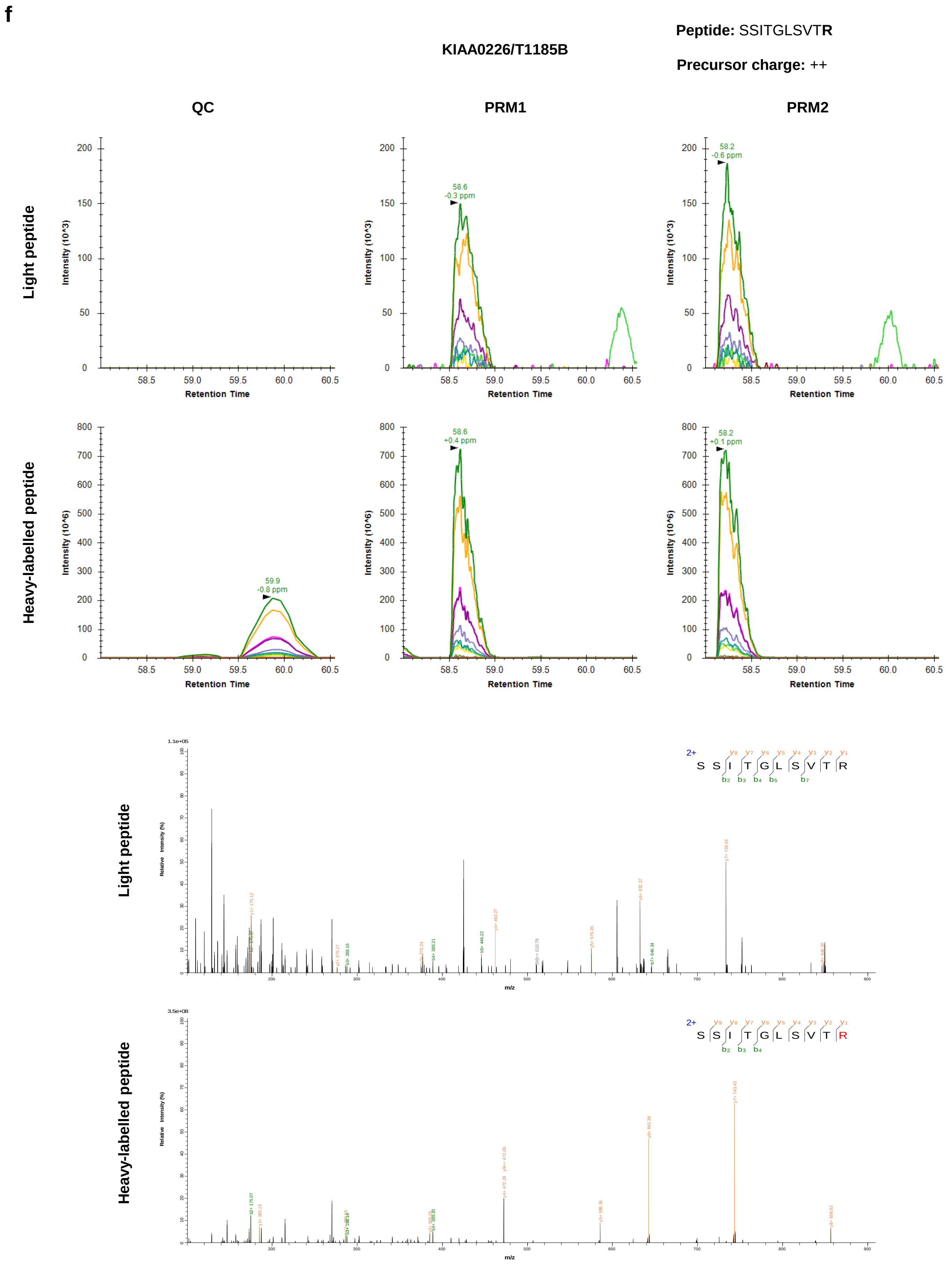
f
KIAA0226/T1185B
Peptide: SSITGLSVTR
Precursor charge: ++
QC
PRM1
PRM2
Light peptide
Heavy-labelled peptide
Intensity (10^3)
Intensity (10^6)
Retention Time
58.6
-0.3 ppm
58.2
-0.6 ppm
59.9
-0.8 ppm
58.6
+0.4 ppm
58.2
+0.1 ppm
1.1e+05
3.5e+08
Relative Intensity (%)
m/z
2+

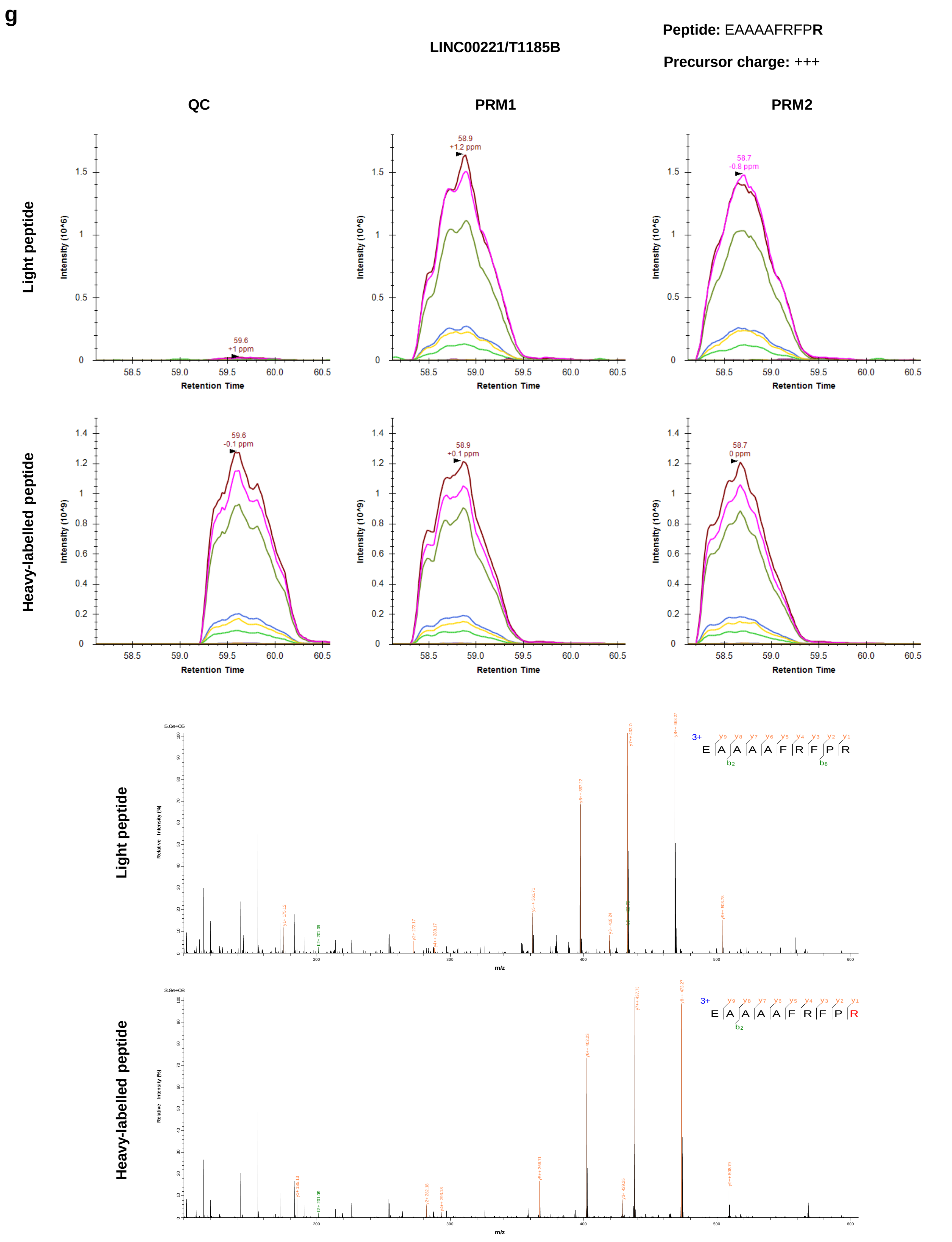
g
LINC00221/T1185B
Peptide: EAAAAFRFPR
Precursor charge: +++
QC
PRM1
PRM2
Light peptide
Heavy-labelled peptide
Intensity (10^6)
Intensity (10^9)
Retention Time
59.6
+1 ppm
58.9
+1.2 ppm
58.7
-0.8 ppm
59.6
-0.1 ppm
58.9
+0.1 ppm
58.7
0 ppm
Light peptide
Heavy-labelled peptide
m/z
3+
E A A A A F R F P R

h

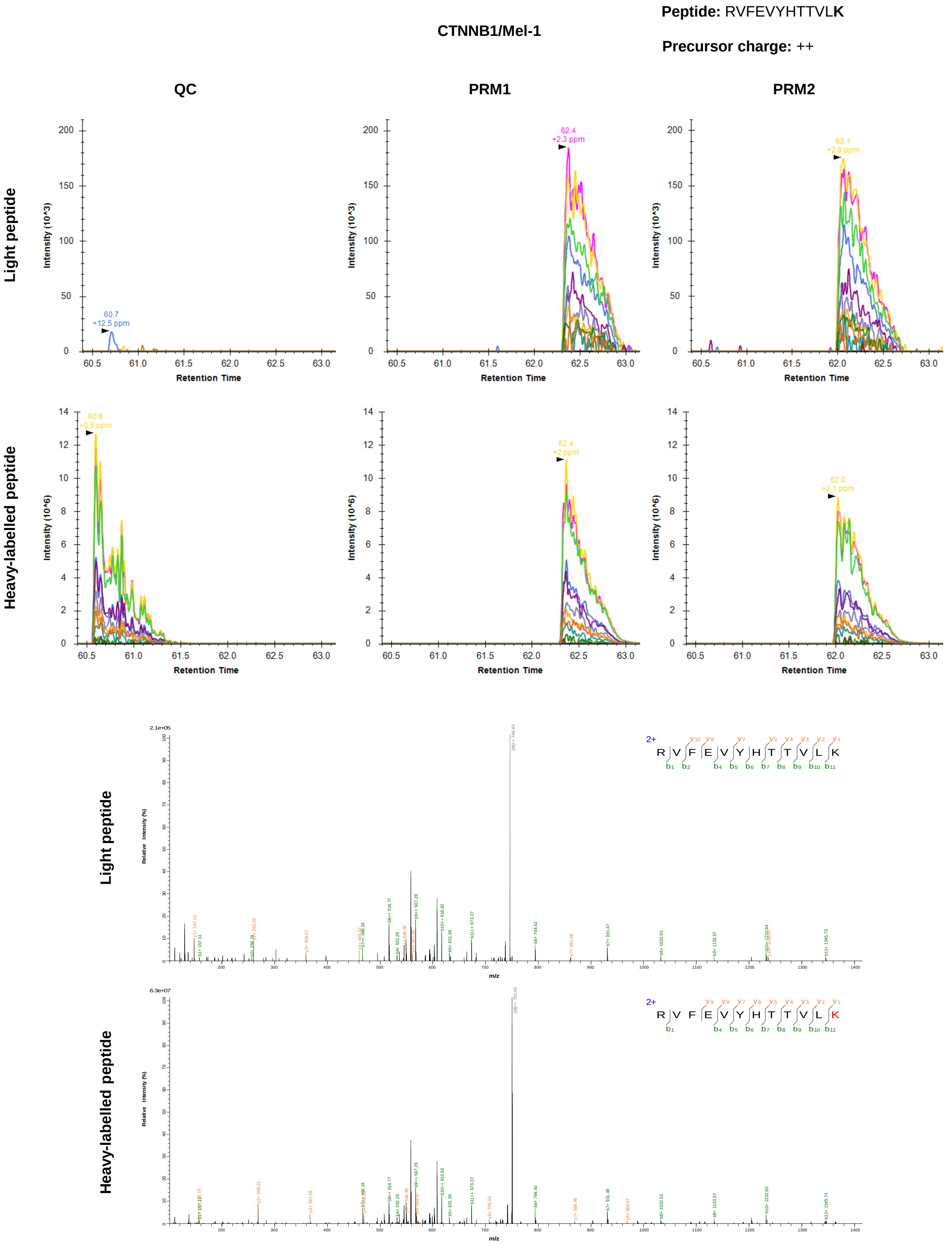
CTNNB1/Mel-1
Peptide: RVFEVYHTTVLK
Precursor charge: ++
QC
PRM1
PRM2
Light peptide
Heavy-labelled peptide
Intensity (10^3)
Intensity (10^6)
Retention Time
60.7
+12.5 ppm
62.4
+2.3 ppm
62.1
+2.8 ppm
60.6
+0.8 ppm
62.4
+2 ppm
62.0
+2.1 ppm
Relative Intensity (%)
m/z
2.1e+05
6.3e+07
2+

i

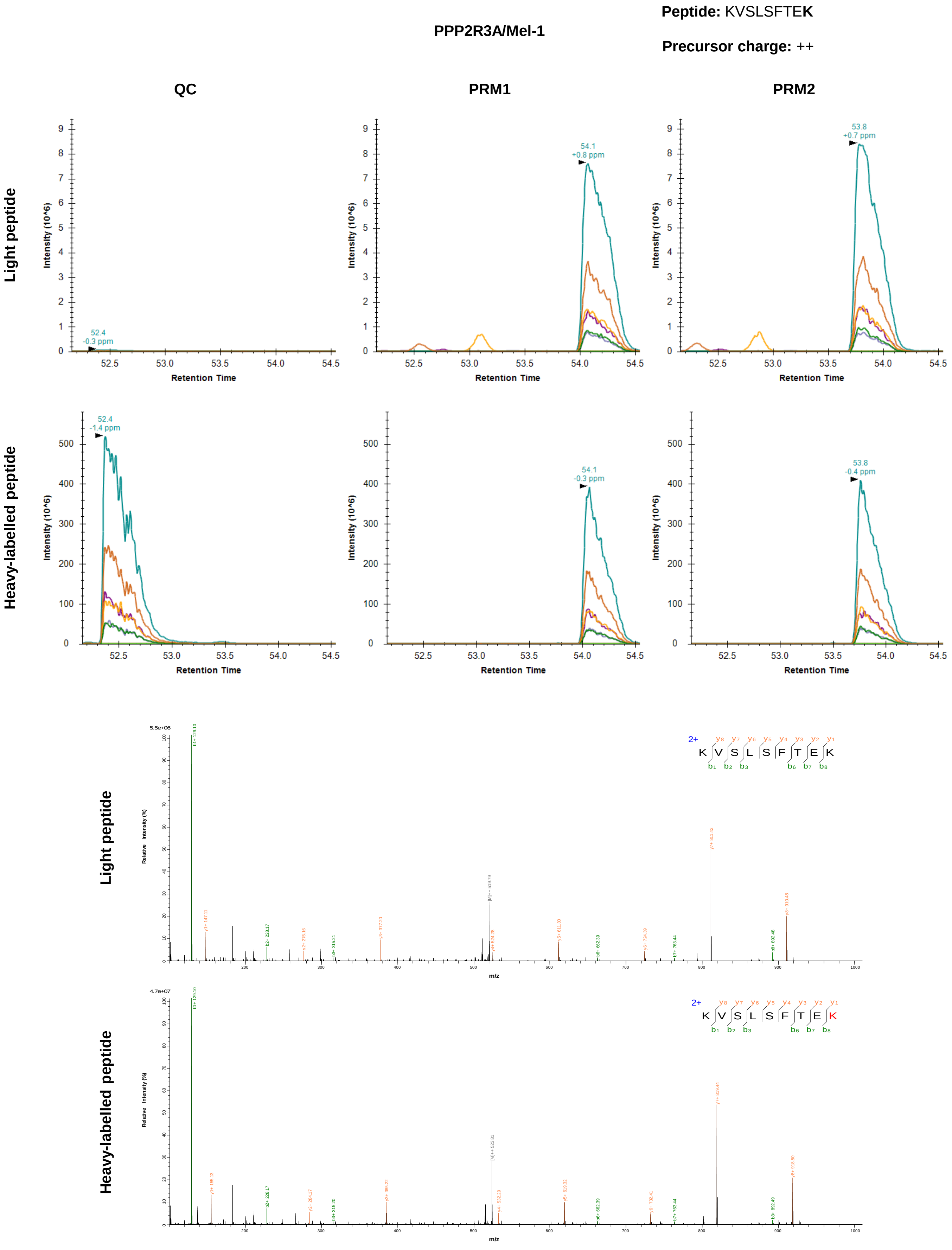
PPP2R3A/Mel-1
Peptide: KVSLSFTEK
Precursor charge: ++
QC
PRM1
PRM2
Light peptide
Heavy-labelled peptide
Intensity (10^6)
Retention Time
52.4
-0.3 ppm
54.1
+0.8 ppm
53.8
+0.7 ppm
52.4
-1.4 ppm
54.1
-0.3 ppm
53.8
-0.4 ppm
5.5e+06
4.7e+07
Relative Intensity (%)
m/z
2+
K V S L S F T E K

j

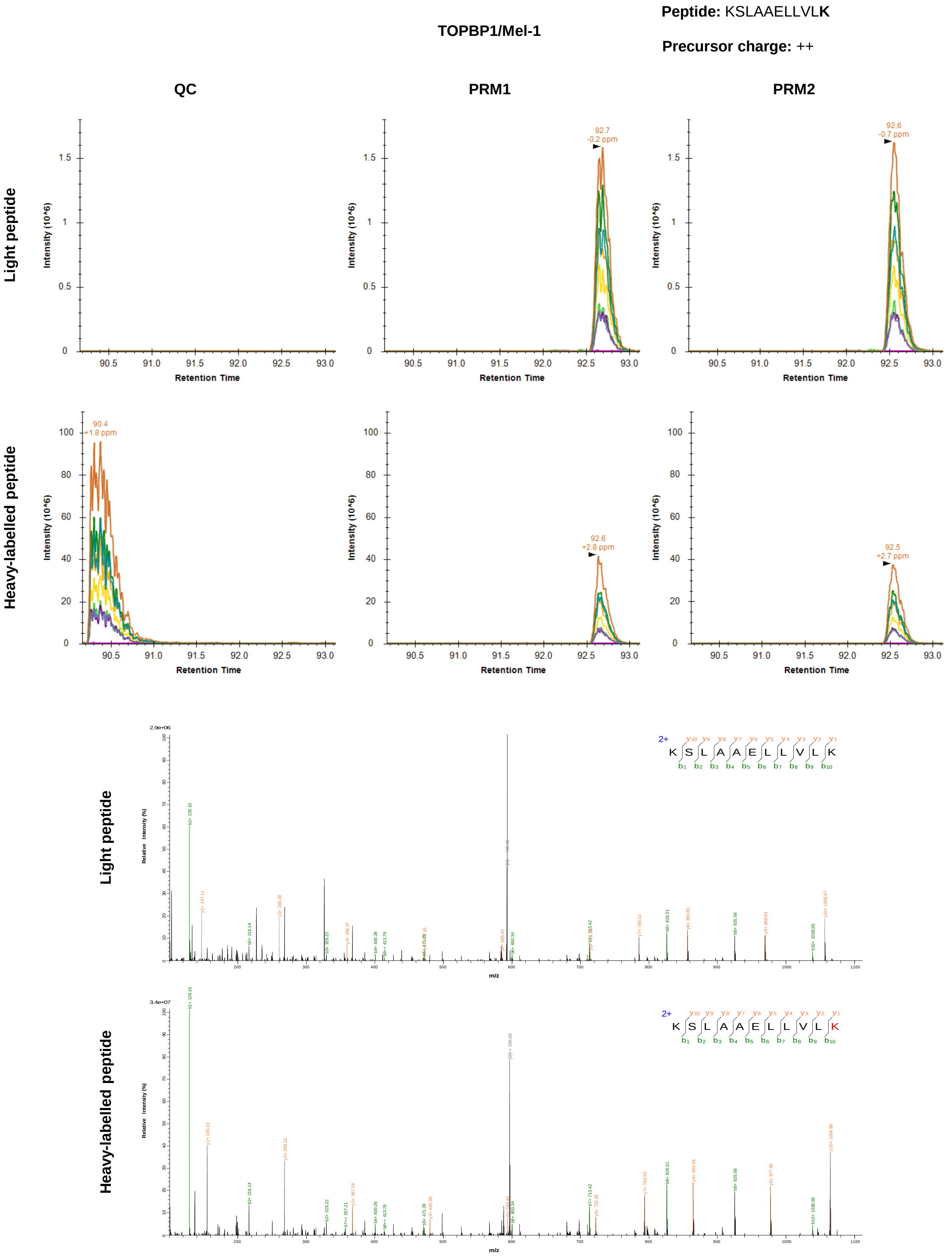

TOPBP1/Mel-1
Peptide: KSLAAELLVLK
Precursor charge: ++
QC
PRM1
PRM2
Light peptide
Heavy-labelled peptide
Intensity (10^6)
Retention Time
92.7
-0.2 ppm
92.6
-0.7 ppm
90.4
+1.8 ppm
92.6
+2.8 ppm
92.5
+2.7 ppm
m/z
Relative Intensity (%)
2.9e+06
3.4e+07

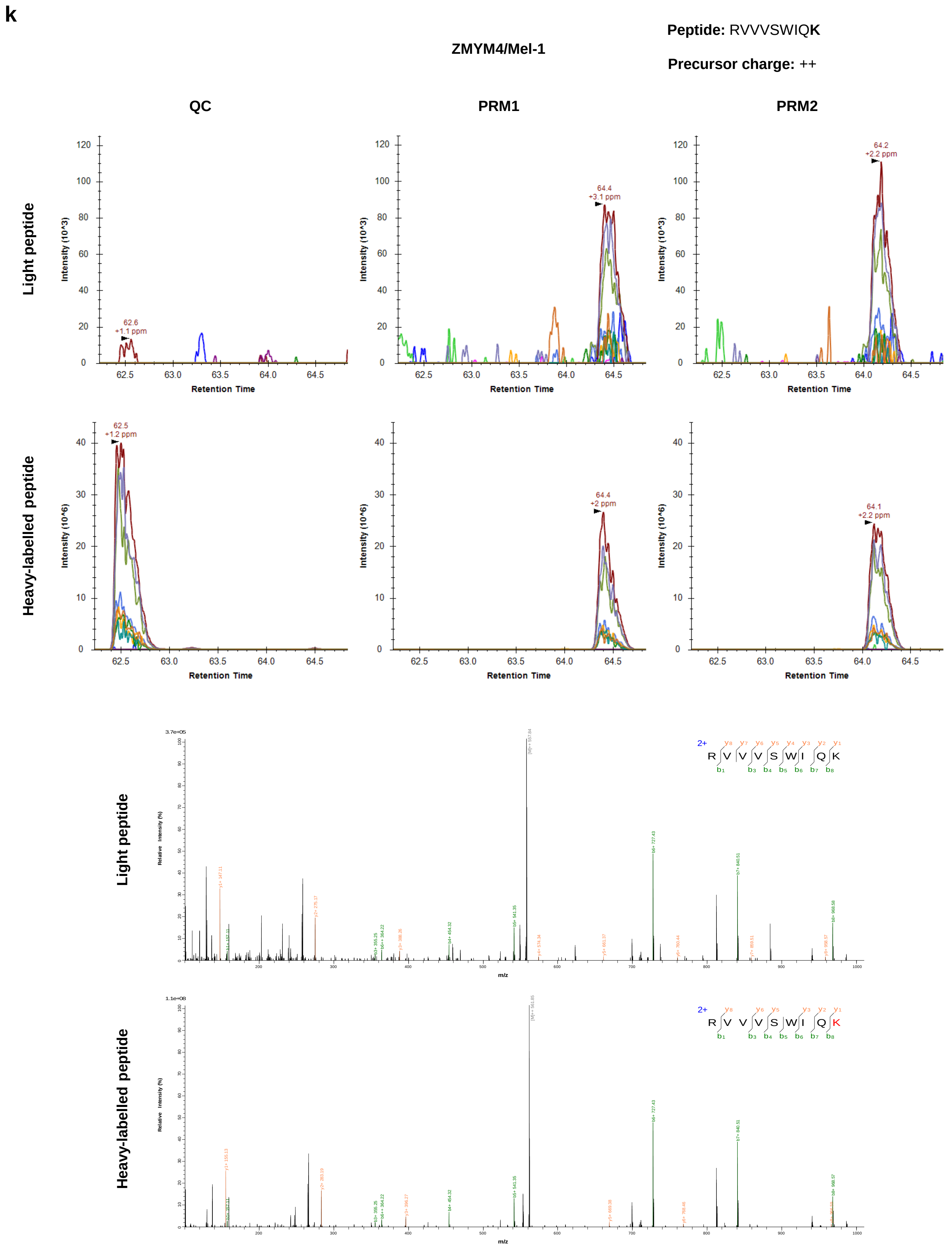
k
ZMYM4/Mel-1
Peptide: RVVVSWIQK
Precursor charge: ++
QC
PRM1
PRM2
Light peptide
Heavy-labelled peptide
Intensity (10^3)
Intensity (10^6)
Retention Time
62.6 +1.1 ppm
64.4 +3.1 ppm
64.2 +2.2 ppm
62.5 +1.2 ppm
64.4 +2 ppm
64.1 +2.2 ppm
3.7e+05
1.1e+08
Relative Intensity (%)
m/z
2+
R V V V S W I Q K

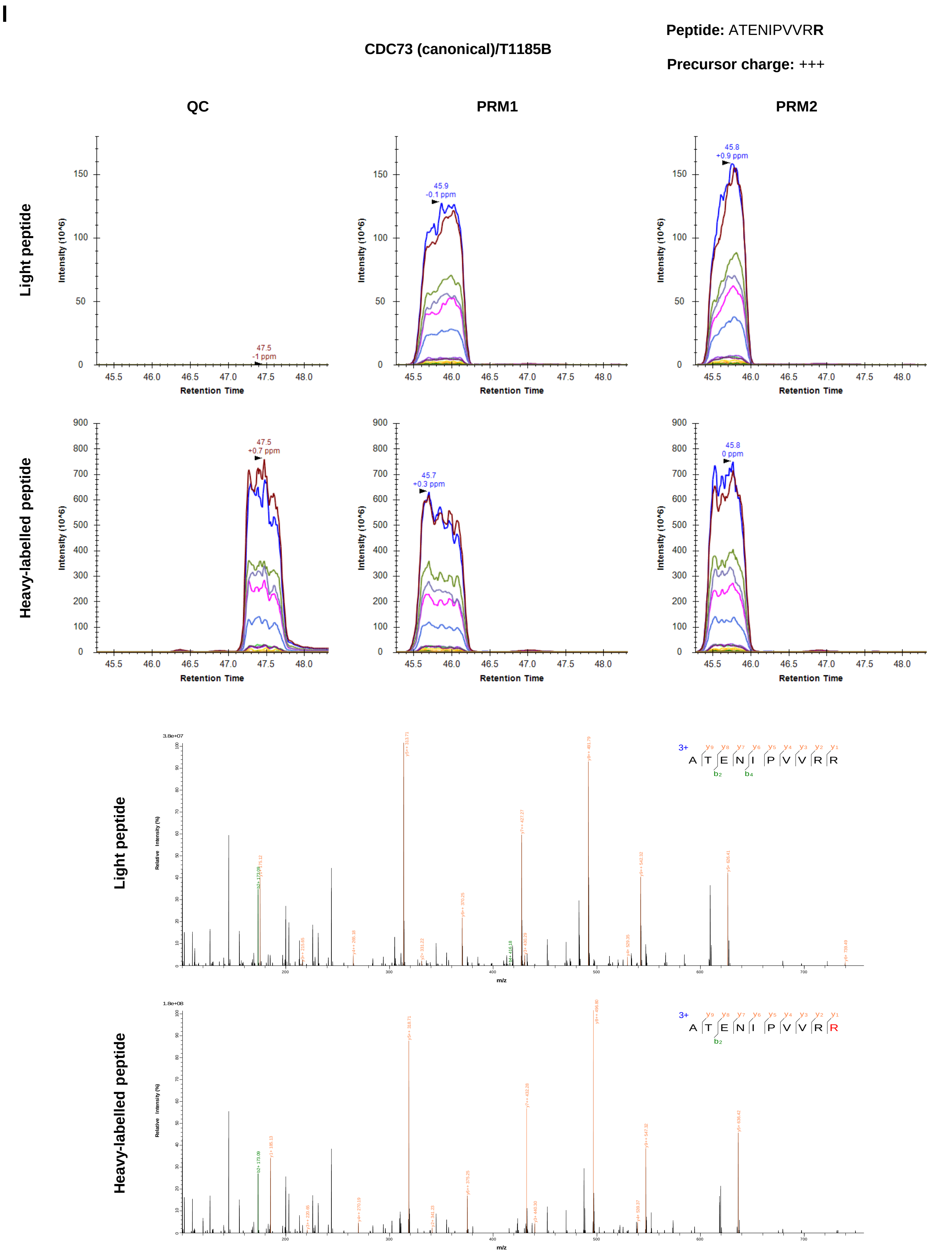
I
CDC73 (canonical)/T1185B
Peptide: ATENIPVVRR
Precursor charge: +++
QC
PRM1
PRM2
Light peptide
Heavy-labelled peptide
Intensity (10^6)
Retention Time
47.5
-1 ppm
45.9
-0.1 ppm
45.8
+0.9 ppm
47.5
+0.7 ppm
45.7
+0.3 ppm
45.8
0 ppm
Light peptide
3.8e+07
Relative Intensity (%)
m/z
3+
A T E N I P V V R R
Heavy-labelled peptide
1.8e+08
Relative Intensity (%)
m/z
3+
A T E N I P V V R R

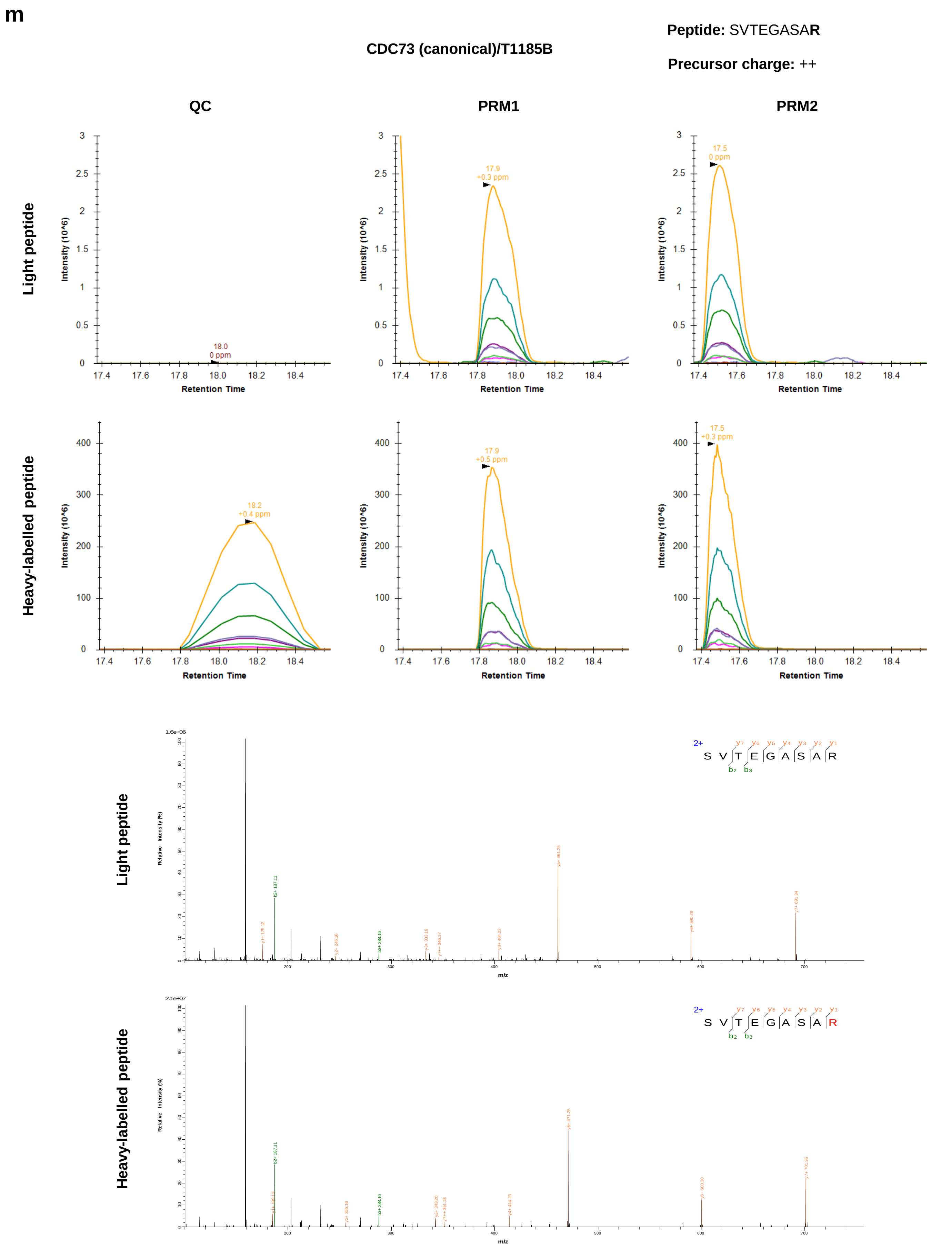
m
CDC73 (canonical)/T1185B
Peptide: SVTEGASAR
Precursor charge: ++
QC
PRM1
PRM2
Light peptide
Heavy-labelled peptide
Intensity (10^6)
Retention Time
18.0
0 ppm
17.9
+0.3 ppm
17.5
0 ppm
18.2
+0.4 ppm
17.9
+0.5 ppm
17.5
+0.3 ppm
Relative Intensity (%)
m/z
1.6e+06
2.1e+07
2+
S V T E G A S A R
y7 y6 y5 y4 y3 y2 y1
b2 b3

Supplementary Figure 4. PRM validation of selected circRNA-derived peptides identified in T1185B and Mel-1 samples. Skyline and pLabel showing the co-elution and MS/MS fragmentation patterns of light (above) and heavy-labelled (below) peptides, respectively. Visualization of MS-detected circRNA-derived peptides from host genes (**a**) *COP1* (*RFWD2*), (**b**) *CDC73*, (**c**) *CTNNB1* (T1185B), (**d**) *CTSB*, (**e**) *GAS7*, (**f**) *KIAA0226*, (**g**) *LINC00221*, (**h**) *CTNNB1* (Mel-1), (**i**) *PPP2R3A*, (**j**) *TOPBP1* and (**k**) *ZMYM4*. (**l**, **m**) Visualization of two MS-detected canonical peptides from CDC73 in T1185B. Corresponding cell line and precursor charge are shown per each peptide. PRM1 and PRM2 represent technical replicates. Heavy labelled amino acid in each synthetic peptide is shown in bold.

a

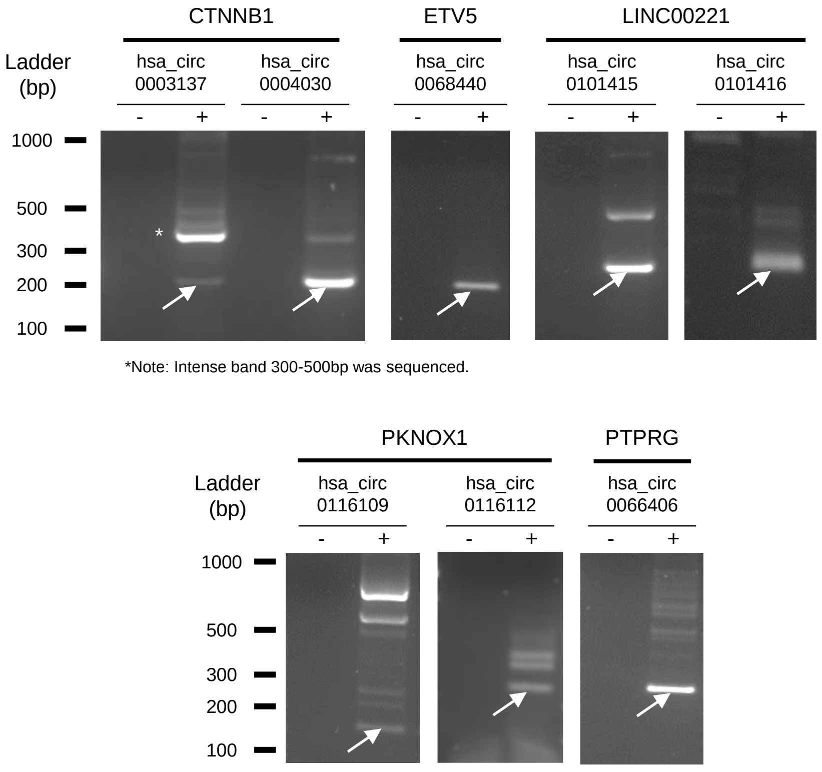

b

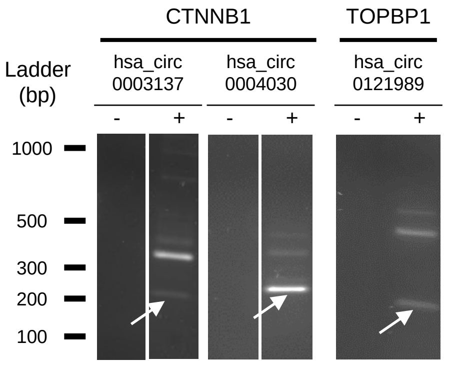

Supplementary Figure 5. Divergent RT-PCR for circRNA validation. (**a**) T1185B cell line. Agarose gel electrophoresis showing the expected size amplicons (arrows). Oligos used for RT-PCR amplification were designed to not overlap the BSJ (region aimed to be sequenced). Expectedly, several unspecificities were observed. RT-PCR amplification of the sequence containing the BSJ of *hsa_circ0003137* showed an intense band (300-500bp). Sanger sequencing showed that the amplification corresponded to *hsa_circ_0004194*. CircRNAs hosted by *CTNNB1*, *ETV5*, *LINC00221*, *PKNOX1* and *PTPRG* genes were validated by divergent RT-PCR followed by Sanger Sequencing. "-": water; "+": T1185B-derived cDNA. (**b**) Mel-1 cell line. Agarose gel electrophoresis showing the expected size amplicons (arrows) for circRNAs hosted by *CTNNB1* and *TOPBP1* genes. Non-adjacent lanes corresponding to the negative controls (water) of the two circRNA amplicons from *CTNNB1* host gene, in Mel-1 cell line, derived from the same gel and they are shown separately. Remaining electrophoresis from T1185B cell line are shown in Fig. 2i (*COP1*/*RFWD2* host gene) and Fig. 3d (*CDC73* host gene). Images were adjusted for better visibility. This experiment was performed once. "-": water; "+": Mel-1-derived cDNA. Source data are provided as a Source Data file.

a

Peptide: RVFEVYHTTVLK
Host gene: CTNNB1
circRNA: hsa_circ_0003137
Expected sequence around the [BSJ]: AGCTTATGGCAACCAAGAAAGCAA[GG]GTATTTGAAGTATACCATACAACT

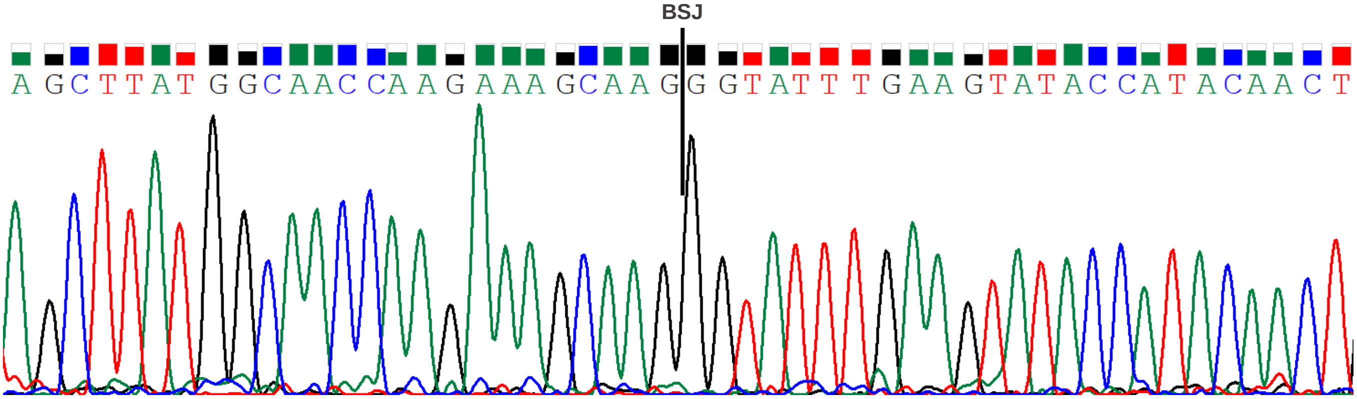

b

Peptide: RVFEVYHTTVLK
Host gene: CTNNB1
circRNA: hsa_circ_0004030
Expected sequence around the [BSJ]: TACTGACCTGTAAATCATCCTTTA[GG]GTATTTGAAGTATACCATACAAC

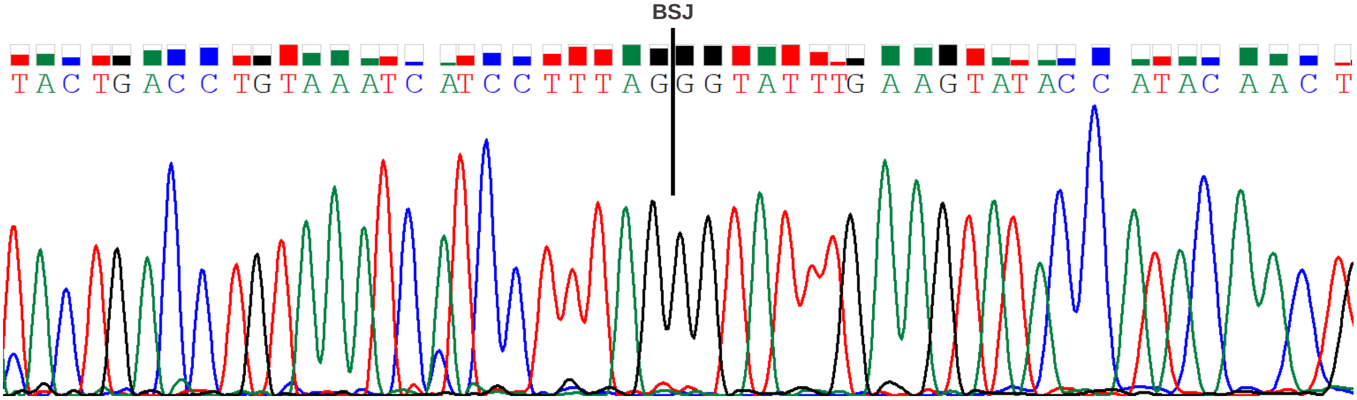

c

Peptide: FVPDFQSDNR
Host gene: ETV5
circRNA: hsa_circ_0068440
Expected sequence around the [BSJ]: GTCCCAGATTTTCAGTCTGATAAC[CG]AGGATCCCTTTTCCCCCAGAAATT

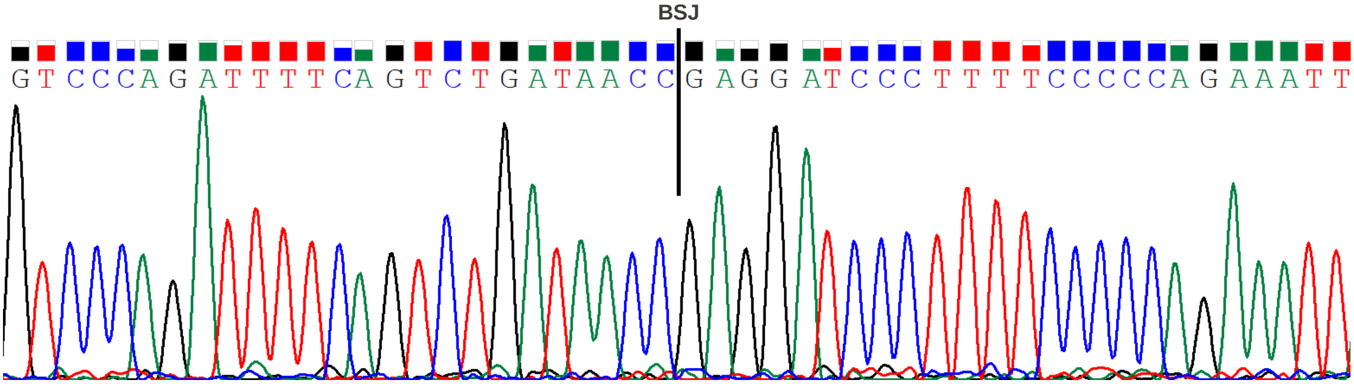

d

Peptide: EAAAAFRFPR (not validated by PRM: AAAAFRFPR)
Host gene: LINC00221
circRNA: hsa_circ_0101415
Expected sequence around the [BSJ]: CCGCCGCTGCCTTCAGGTTTCCGA[GG]GACTTTTGTGTCTGTGCTCACATA

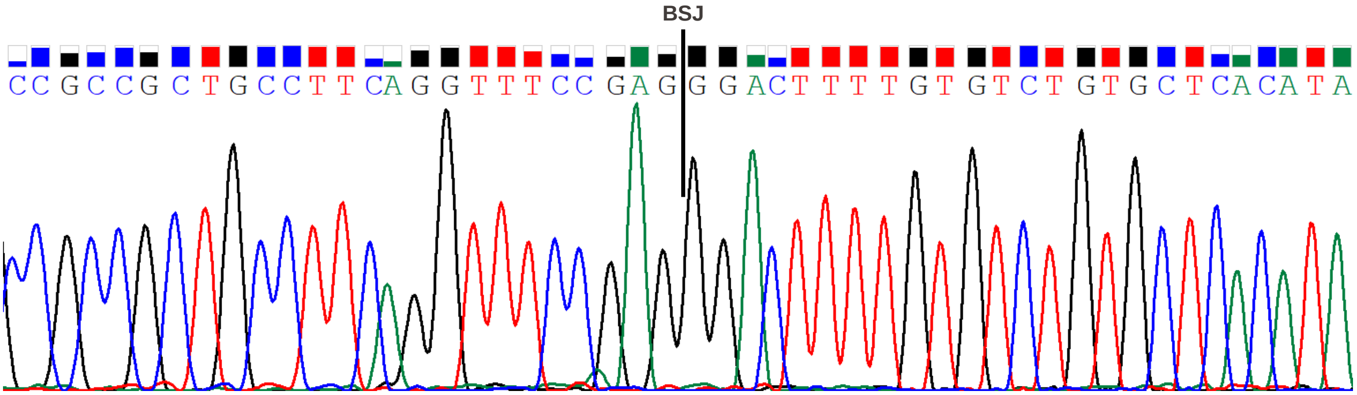

e

Peptide: EAAAAFRFPR (not validated by PRM: AAAAFRFPR)
Host gene: LINC00221
circRNA: hsa_circ_0101416
Expected sequence around the [BSJ]: CCGCCGCTGCCTTCAGGTTTCCGA[GG]ATGTGAGGAAGTAGAACCACACAC

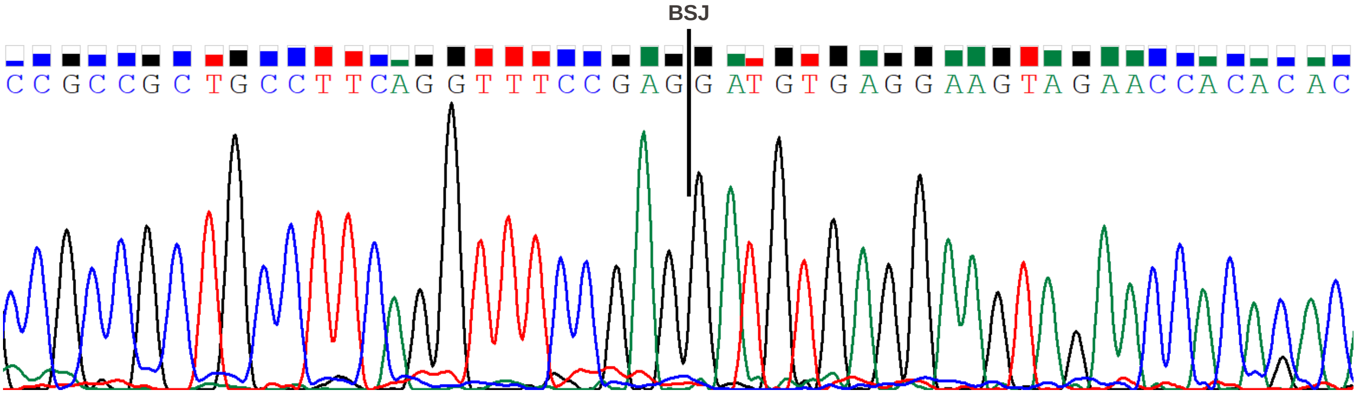

f

Peptide: QTPFAFHPR
Host gene: PKNOX1
circRNA: hsa_circ_0116109
Expected sequence around the [BSJ]: CCCGTACTCACCAGTGCAGTCCCA[GA]CACCATTCGCTTTTCACCCAAGAT

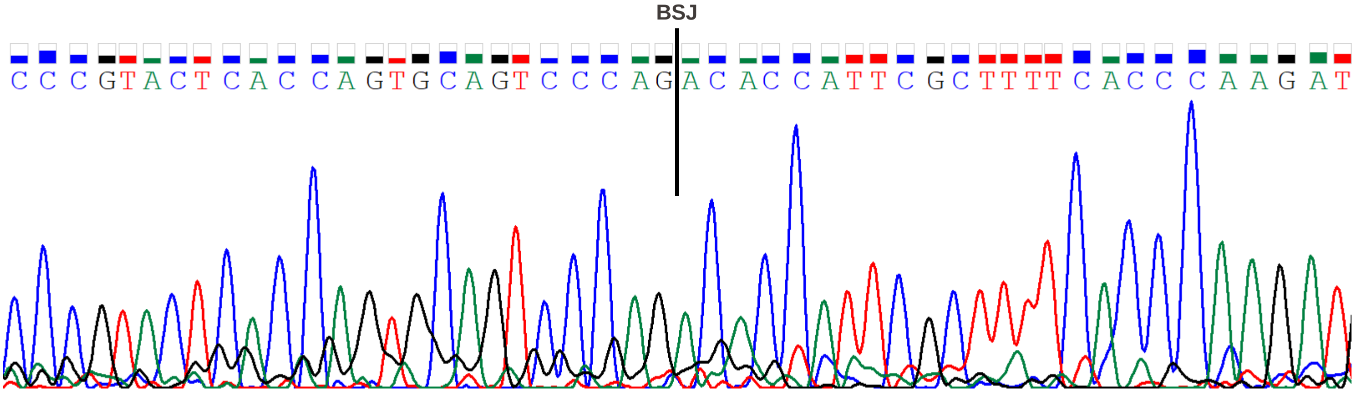

g

Peptide: QTPFAFHPR
Host gene: PKNOX1
circRNA: hsa_circ_0116112
Expected sequence around the [BSJ]: CGGGGCCACCCTGGCGGTGCAGCA[GA]CACCATTCGCTTTTCACCCAAGAT

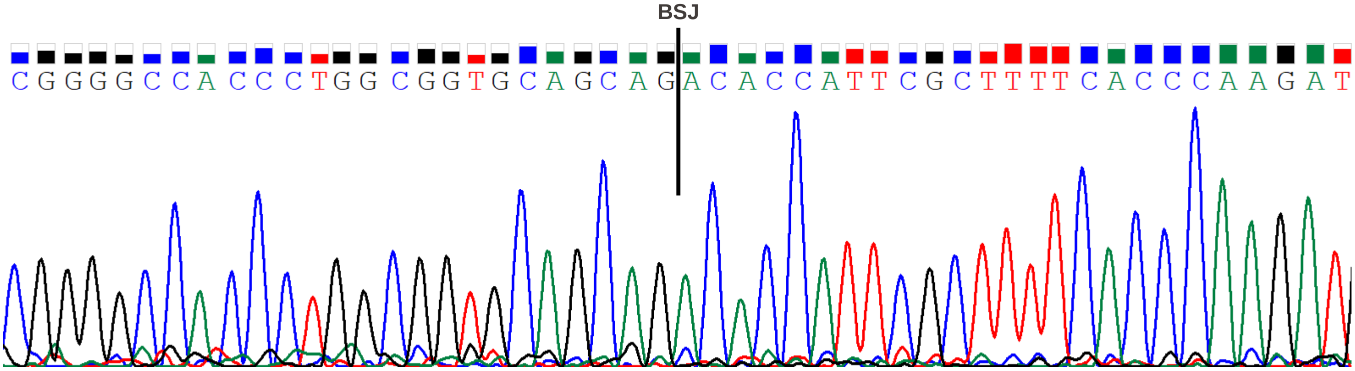

h

Peptide: NTASMAGGFLLR
Host gene: PTPRG
circRNA: hsa_circ_0066406
Expected sequence around the [BSJ]: CAATGGCAGGAGGTTTCCTGTTGA[GG[TGCCTATGGTCCTGAGCACTGGGT

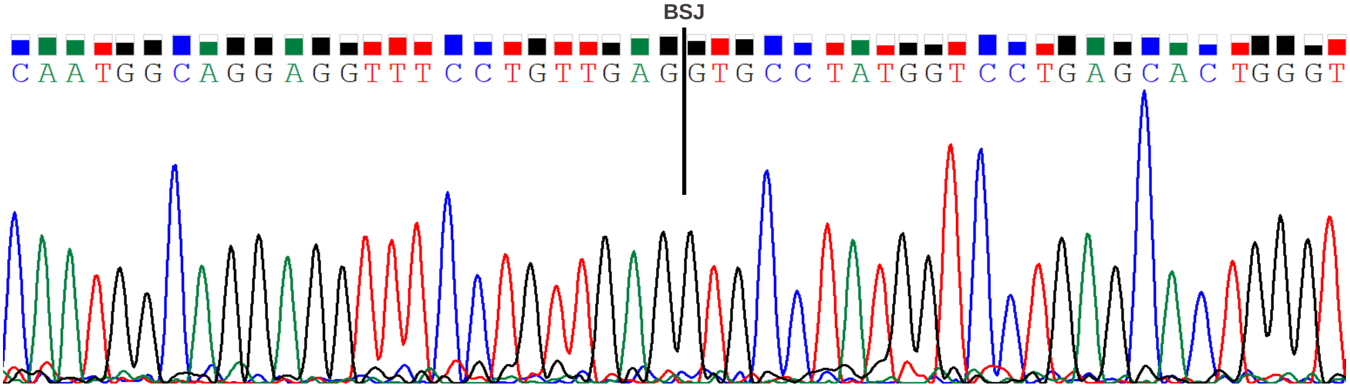

i

Peptide: RVFEVYHTTVLK
Host gene: CTNNB1
circRNA: hsa_circ_0003137
Expected sequence around the [BSJ]: AGCTTATGGCAACCAAGAAAGCAA[GG]GTATTTGAAGTATACCATACAACT

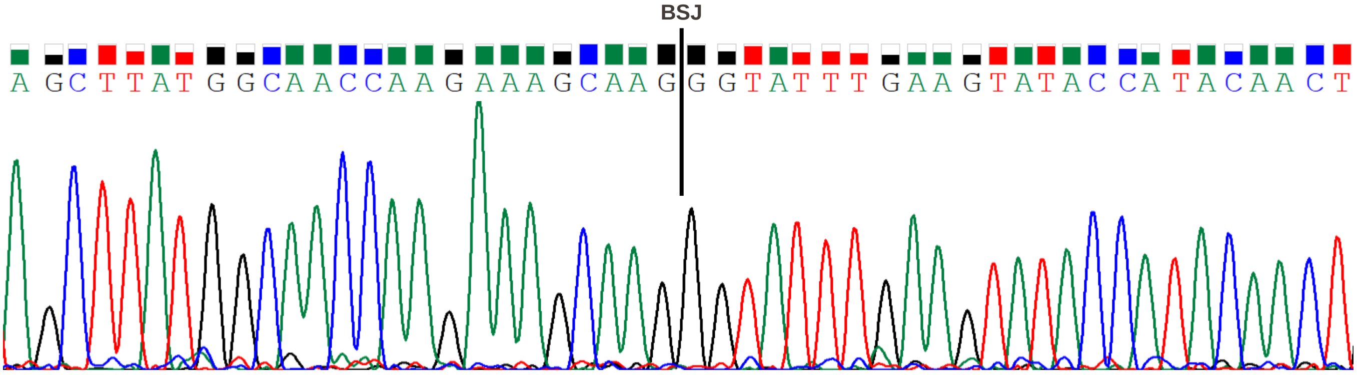

j

Peptide: RVFEVYHTTVLK
Host gene: CTNNB1
circRNA: hsa_circ_0004030
Expected sequence around the [BSJ]: TACTGACCTGTAAATCATCCTTTA[GG]GTATTTGAAGTATACCATACAAC

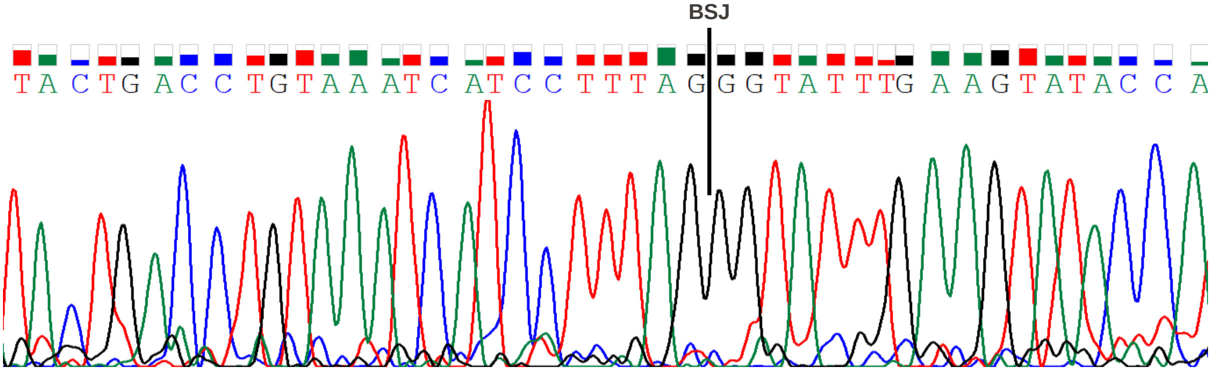

k

Peptide: KSLAAELLVLK
Host gene: TOPBP1
circRNA: hsa_circ_0121989
Expected sequence around the [BSJ]: CACCTCATTGTGCAAGAACCAAAA[GC]TTGGCTGCAGAATTGTTGGTCCTC

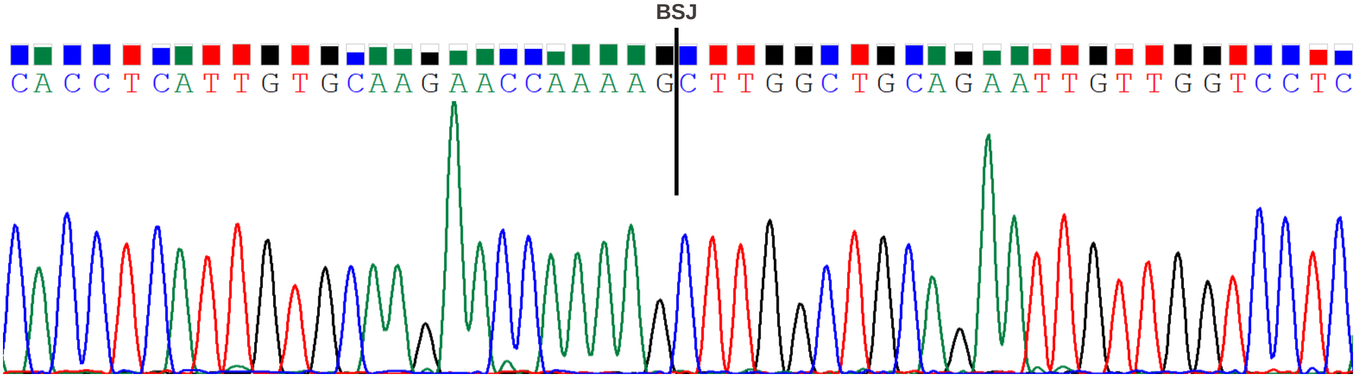

l

Extra band obtained during amplification of hsa_circ0003137:
Host gene: CTNNB1
circRNA: hsa_circ_0004194 (known circRNA)
Expected sequence around the [BSJ]: AATAAGCCGGCTATTGTAGAAGCT[GG]GTATTTGAAGTATACCATACAACT

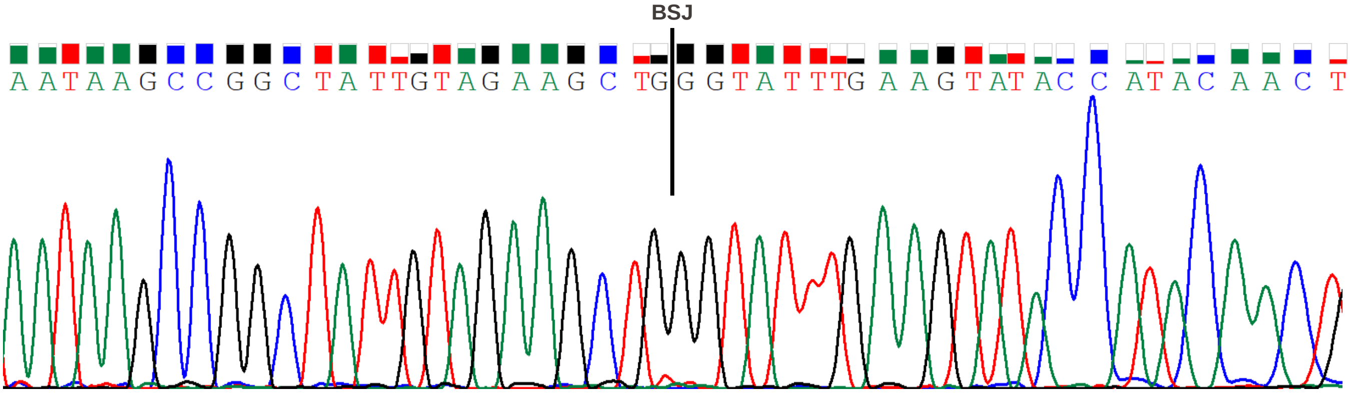

Supplementary Figure 6. Sanger Sequencing validation in T1185B and Mel-1 cell lines. Following electrophoresis, bands with the expected size were purified and DNA was analyzed by direct Sanger Sequencing, validating the sequence overlapping the BSJ of (**a**) *hsa_circ_0003137* (*CTNNB1* in T1185B), (**b**) *hsa_circ_0004030* (*CTNNB1* in T1185B), (**c**) *hsa_circ_0068440* (*ETV5*), (**d**) *hsa_circ_0101415* (*LINC00221*), (**e**) *hsa_circ_0101416* (*LINC00221*), (**f**) *hsa_circ_0116109* (*PKNOX1*), (**g**) *hsa_circ_0116112* (*PKNOX1*), (**h**) *hsa_circ_0066406* (*PTPRG*), (**i**) *hsa_circ_0003137* (*CTNNB1* in Mel-1), (**j**) *hsa_circ_0004030* (*CTNNB1* in Mel-1), (**k**) *hsa_circ_0121989* (*TOPBP1* in Mel-1) and (**l**) *hsa_circ_0004194* (*CTNNB1* extra band in T1185B). Remaining Sanger Sequencing chromatograms from T1185B cell line are shown in Fig. 2j (*hsa_circ_0015366* and *hsa_circ_0111262*, *COP1*/*RFWD2*) and Fig. 3e (*hsa_circ_0111569*, *CDC73*).

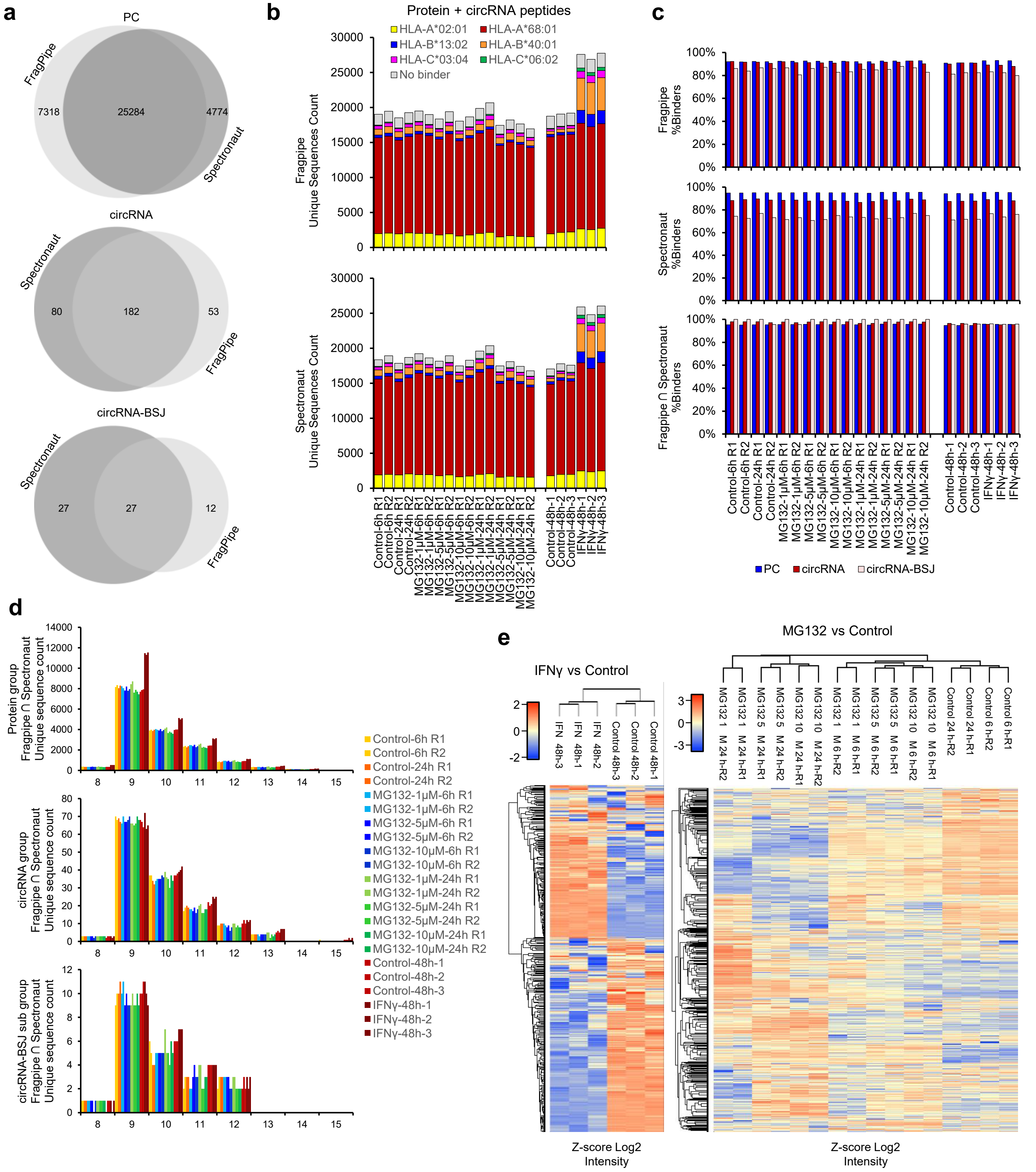

Supplementary Figure 7. IFNg and MG132 treatments in T1185B cell line. (**a**) Venn diagrams show the intersection between the unique peptide sequences detected in FragPipe (group-specific FDR 0.03) and Spectronaut (FDR 0.01), using a hybrid DIA approach. Illustration was done for PC, circRNA, and circRNA-BSJ groups. (**b**) Total number of unique peptides identified by FragPipe (top) and Spectronaut (bottom) in each sample, colored based on their predicted HLA restriction. (**c**) Percentage (%) of peptides predicted as HLA binders (% rank < 2) in each sample, using NetMHCpan, annotated in the PC, circRNA, and circRNA-BSJ groups, for FragPipe (top), Spectronaut (middle) and their intersection (bottom). (**d**) Length distribution of the common identified peptides in FragPipe and Spectronaut among PC, circRNA, and circRNA-BSJ groups. (**e**) Hierarchical clustering heatmaps showing the Z-score Log2 intensity of the peptides following IFNg or MG132 treatments. Source data are provided as a Source Data file.

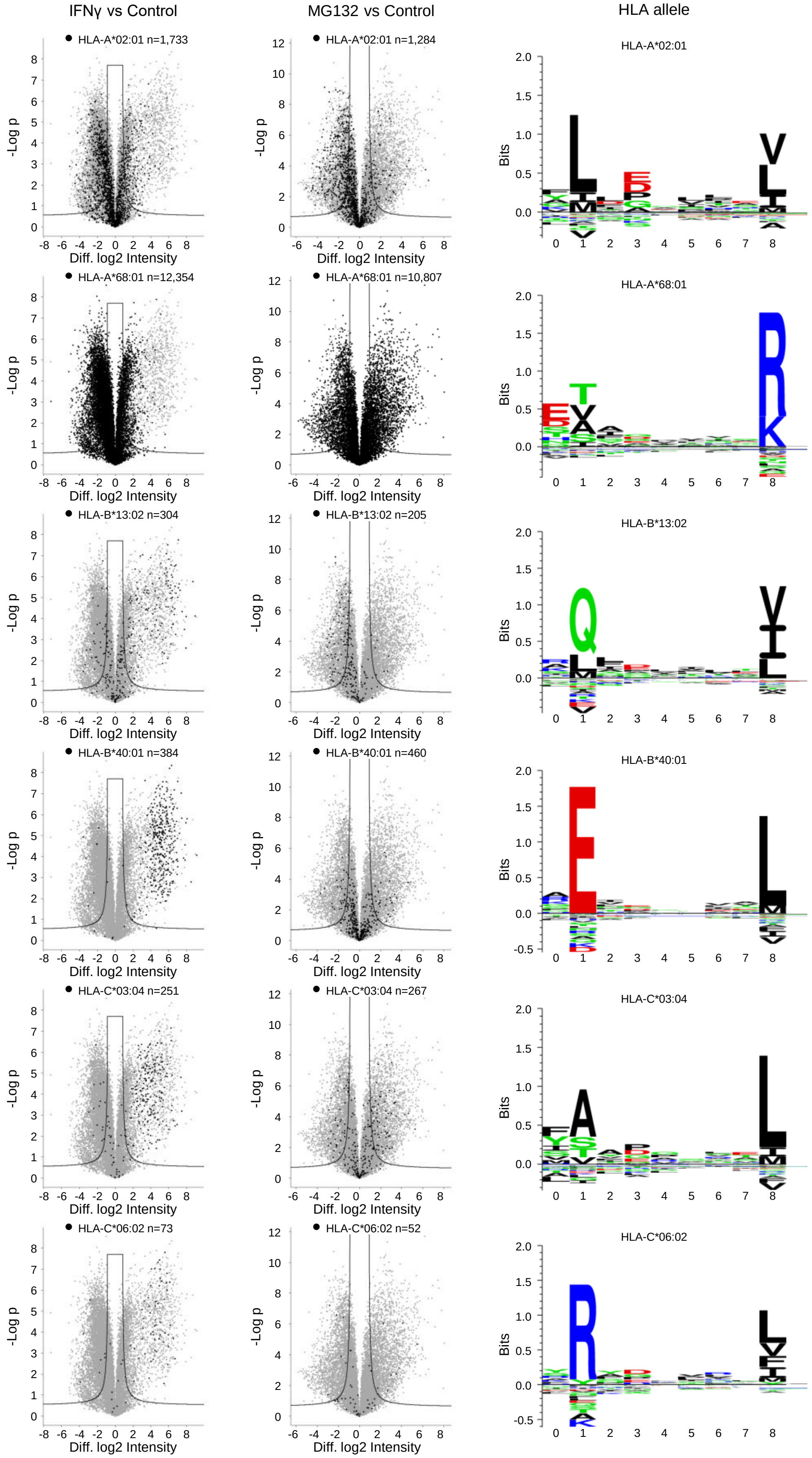

Supplementary Figure 8. Effect of IFNγ and MG132 in the intensity of peptides per binder allele. Volcano plots highlighting the peptides associated to each HLA-I allele in black (best binder allele). HLA allele binding motifs based on Mass-Spectrometry Eluted Ligands (EL) are shown on the right of the volcano plots (derived from NetMHCpan - 4.1). Source data are provided as a Source Data file.

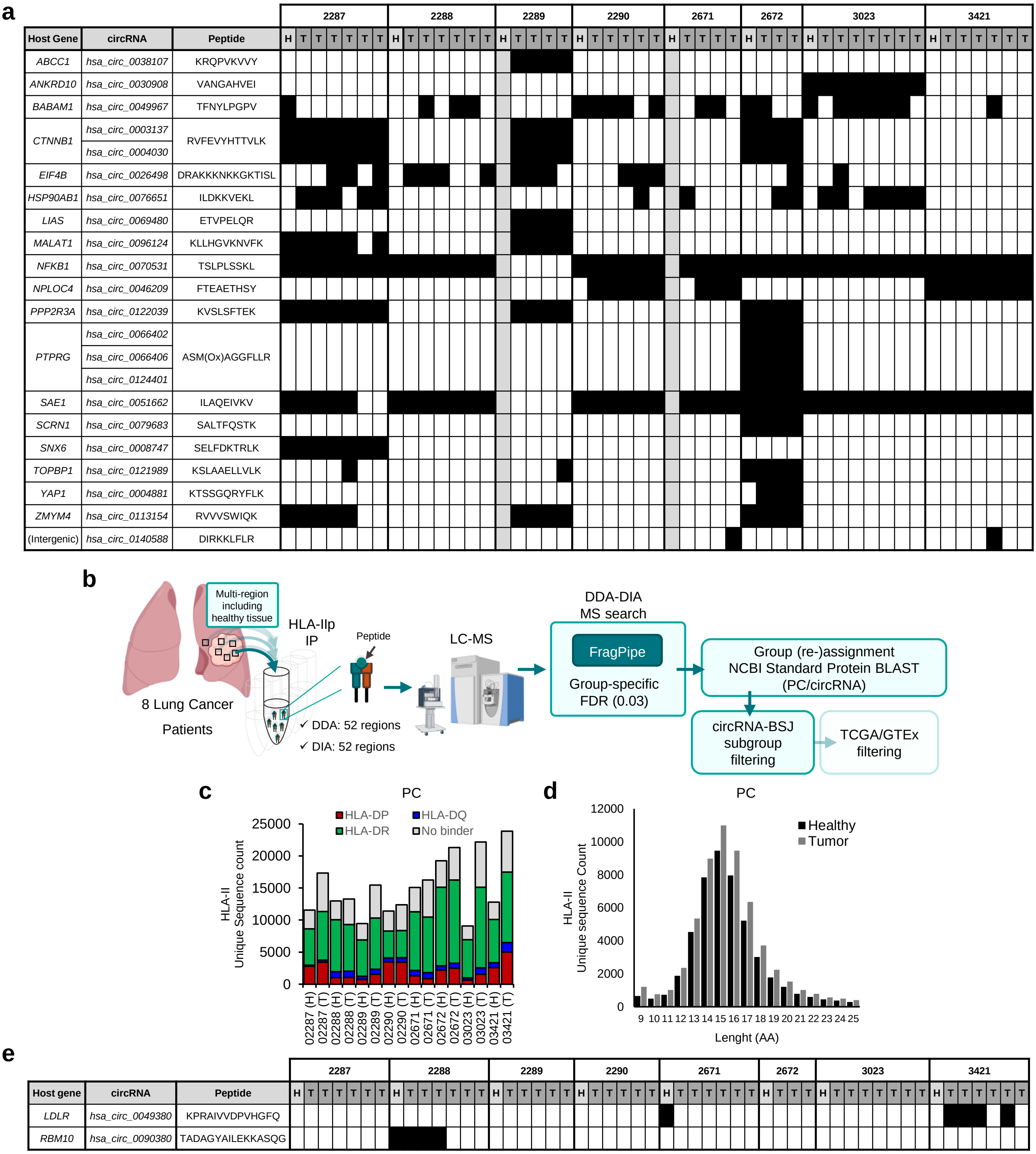

Supplementary Figure 9. Detection of circRNA-BSJ derived peptides across multi-region lung cancer and healthy tissues in eight patients. (**a**) Identification of HLA-I peptides across the samples. (**b**) Workflow used to explore HLA-II immunopeptidomics DDA and DIA MS data of 8 lung cancer patients, including 52 tumoral and healthy matched tissues. MS raw files were analyzed by a DDA-DIA approach with FragPipe, applying a group-specific FDR threshold of 0.03 for protein-derived and circRNA-derived peptides groups. circRNA-derived peptides were blasted against a larger Reference Proteins database to re-assign matching peptides to the protein (PC) group. Schema was partially created with BioRender.com. (**c**) Unique peptide count and (**d**) length distribution of the protein-derived HLA-II immunopeptidome in healthy (H) and tumor (T) tissues. (**e**) Identification of circRNA-BSJ derived HLA-II peptides across samples. Black square: detected peptide; white square: not detected peptide; grey square: no data. Source data are provided as a Source Data file.

Supplementary Table 1. List of the synthetic heavy-labelled peptides correspondent to the validated targets.

Host gene	Peptide Sequence	Nterm	CTerm	MW	Cell line	#Cells	Spike-in (pmol/uL)
CDC73	TTENIPVVR**R**	[H]	[OH]	1194.284	T1185B	2x10^8 cells	1
CTNNB1	RVFEVYHTTVL**K**	[H]	[OH]	1499.6896	T1185B	1x10^8 cells	1
					Mel-1	1x10^8 cells	1
CTSB	STNGIPRG**R**	[H]	[OH]	967.06	T1185B	1x10^8 cells	0.5
GAS7	EAWNGPPSAG**R**	[H]	[OH]	1151.1312	T1185B	1x10^8 cells	0.5
KIAA0226	SSITGLSVT**R**	[H]	[OH]	1030.0738	T1185B	1x10^8 cells	0.5
LINC00221	EAAAAFRFP**R**	[H]	[OH]	1145.3	T1185B	1x10^8 cells	0.5
RFWD2	DLYNGSSIVS**R**	[H]	[OH]	1220.31	T1185B	2x10^8 cells	1
PPP2R3A	KVSLSFTE**K**	[H]	[OH]	1046.1452	Mel-1	1x10^8 cells	1
TOPBP1	KSLAAELLVL**K**	[H]	[OH]	1192.4211	Mel-1	1x10^8 cells	1
ZMYM4	RVVVSWIQ**K**	[H]	[OH]	1122.2923	Mel-1	1x10^8 cells	1
CDC73 (linear mRNA)	ATENIPVVR**R**	[H]	[OH]	1164.2577	T1185B	2x10^8 cells	1
	SVTEGASA**R**	[H]	[OH]	886.8448	T1185B	1x10^8 cells	1

Note: Position of the heavy-labelled amino acid is indicated in bold.

Supplementary Table 2. List of oligos used for divergent PCR/ Sanger Sequencing.

Host gene	circRNA	Forward Oligo	Reverse Oligo	PCR product (bp)	Sequencing Oligo
CDC73	*hsa_circ_0111569*	GCACCTGTGGATCCCACTTT	ATACTTGCCGATGTTGACGC	239	ATACTTGCCGATGTTGACGC
CTNNB1	*hsa_circ_0003137*	GGAGCTAAAATGGCAGTGCG	ACTCCATCAAATCAGCTTGAGTA	211	GGAGCTAAAATGGCAGTGCG
CTNNB1	*hsa_circ_0004030*	CCACCCTGGTGCTGACTATC	ACTCCATCAAATCAGCTTGAGTA	209	ACTCCATCAAATCAGCTTGAGTA
ETV5	*hsa_circ_0068440*	GGACACAGATCTGGCTCACG	ATGGTGCTTTCAGCGTCTCT	204	GGACACAGATCTGGCTCACG
LINC00221	*hsa_circ_0101415*	TATGTGGTACAGGGTTGGGC	CAATGGGACTCGCCTTCAGT	248	TATGTGGTACAGGGTTGGGC
LINC00221	*hsa_circ_0101416*	AGCGGGGACTTCCTTTATGTG	GAATACAAGGCCGGGTCAGG	239	AGCGGGGACTTCCTTTATGTG
PKNOX1	*hsa_circ_0116109*	TCTGCAGTCGATACATTGCTTG	TGCATCTGTTGCCCATCTTGA	210	TGCATCTGTTGCCCATCTTGA
PKNOX1	*hsa_circ_0116112*	CCACGCCCGTGAACATGAAC	GCATCGGGTTCAGAGCAGTT	223	GCATCGGGTTCAGAGCAGTT
PTPRG	*hsa_circ_0066406*	GCTGGTCTACCTGGCAGATTC	TCGAAGCCATCGAGTTGCAG	244	TCGAAGCCATCGAGTTGCAG
RFWD2	*hsa_circ_0015366*	CTGCAAGCCAGTTGGATGAA	TGGATCTGTTCCAGTTGCTCTC	210	CTGCAAGCCAGTTGGATGAA
RFWD2	*hsa_circ_0111262*	CAGATGACAGTCGAACTGCAAG	TCAAGACTAGGATGCCCCGC	194	CTGCAAGCCAGTTGGATGAA
TOPBP1	*hsa_circ_0121989*	GTGTGACTGGCTTATGTGGC	GATGTTCGGCTCTTGGGACA	205	GTGTGACTGGCTTATGTGGC
