# Supplementary material for: Immunopeptidomics-based identification of naturally presented non-canonical circRNA-derived peptides
Source: Nat Commun. 2024 Mar 15;15:2357. doi: 10.1038/s41467-024-46408-3 (PMC10943130; doi:10.1038/s41467-024-46408-3)
Supplement: Supplementary file 3 — Description of Additional Supplementary Files [file 41467_2024_46408_MOESM3_ESM.pdf]

Description of Additional Supplementary Files

Supplementary Data Legends

Supplementary Data 1: CircRNA-derived peptides, overlapping the BSJ encoding region, detected in T1185B upon MG312 and IFNγ treatments.

Supplementary Data 2: GOBP, GOCC and KEGG presentation enrichment analysis of differentially presented peptides upon IFNγ treatment. Two-sided Wilcoxon-Mann-Whitney test, 1D annotation enrichment, IFNγ versus Control, using Student's t-test difference and a Benjamini-Hochberg FDR threshold value of 1E-4 (FDR=0.02 for HLA-alleles).

Supplementary Data 3: KEGG and GOBP presentation enrichment analysis of differentially presented peptides upon MG132 treatment. Two-sided Wilcoxon-Mann-Whitney test, 1D annotation enrichment, 1D annotation enrichment, MG132 versus Control, using Student's t-test difference and a Benjamini-Hochberg FDR threshold value of 1E-4 (FDR=0.02 for HLA-alleles).

Supplementary Data 4: HLA-I peptide binding prediction in the Lung Cohort (NetMHCpan - 4.1).

Supplementary Data 5: HLA-II circRNA-derived peptides, overlapping the BSJ encoding region, detected in the lung cohort (eight patients with matching healthy tissue) through DDA-DIA FragPipe Analysis with group-specific FDR calculation.

Supplementary Data 6: HLA-II peptide binding prediction in the Lung Cohort (NetMHCIIpan - 4.1).

Supplementary Data 7: List of raw files used in each MS search analysis.
