# Supplementary material for: Immunopeptidomics-based identification of naturally presented non-canonical circRNA-derived peptides
Source: Nat Commun. 2024 Mar 15;15:2357. doi: 10.1038/s41467-024-46408-3 (PMC10943130; doi:10.1038/s41467-024-46408-3)
Supplement: Supplementary file 11 — Reporting Summary [file 41467_2024_46408_MOESM11_ESM.pdf]

nature portfolio

Corresponding author(s): Dr. Michal Bassani-Sternberg

Last updated by author(s): Dec 10, 2023

Reporting Summary

Nature Portfolio wishes to improve the reproducibility of the work that we publish. This form provides structure for consistency and transparency in reporting. For further information on Nature Portfolio policies, see our Editorial Policies and the Editorial Policy Checklist.

Statistics

For all statistical analyses, confirm that the following items are present in the figure legend, table legend, main text, or Methods section.

n/a	Confirmed	
☐	☒	The exact sample size (*n*) for each experimental group/condition, given as a discrete number and unit of measurement
☐	☒	A statement on whether measurements were taken from distinct samples or whether the same sample was measured repeatedly
☐	☒	The statistical test(s) used AND whether they are one- or two-sided<br>*Only common tests should be described solely by name; describe more complex techniques in the Methods section.*
☒	☐	A description of all covariates tested
☐	☒	A description of any assumptions or corrections, such as tests of normality and adjustment for multiple comparisons
☐	☒	A full description of the statistical parameters including central tendency (e.g. means) or other basic estimates (e.g. regression coefficient) AND variation (e.g. standard deviation) or associated estimates of uncertainty (e.g. confidence intervals)
☐	☒	For null hypothesis testing, the test statistic (e.g. *F*, *t*, *r*) with confidence intervals, effect sizes, degrees of freedom and *P* value noted<br>*Give P values as exact values whenever suitable.*
☒	☐	For Bayesian analysis, information on the choice of priors and Markov chain Monte Carlo settings
☒	☐	For hierarchical and complex designs, identification of the appropriate level for tests and full reporting of outcomes
☒	☐	Estimates of effect sizes (e.g. Cohen's *d*, Pearson's *r*), indicating how they were calculated

Our web collection on statistics for biologists contains articles on many of the points above.

Software and code

Policy information about availability of computer code

Data collection	No software was used for data collection.
Data analysis	Softwares used in this study are publicly available and stated in the Methods section where applicable.<br>-PRM: Skyline v20.2.0.343 (64-bit), Proteowizard v3.0.21069, pLabel v2.4.3<br>-HLA typing: Assign TruSight software v2.1<br>-MS analyses: MaxQuant v2.1.0.0, Comet 2019.01 rev. 4, NewAnce v1.6 (see below), PDV 1.7.4, PDV 1.8.2, FragPipe v.19.2-build39, FragPipe v.20, Spectronaut v.18.4<br>-HLA-peptide binding prediction: NetMHCpan4.1<br>-General: Microsoft Excel, Perseus 1.5.5.3, GraphPad Prism 9.5.1, DeepVenn (https://www.deepvenn.com), and Intervene (https://intervene.shinyapps.io/intervene/)<br>-NewAnce: The NewAnce code is available on the following GitHub link: https://github.com/bassanilab/NewAnce<br>-CircRNA_MS_ref_fasta: code required to generate the circRNA-derived BSJ-ORF fasta reference and to adapt the concatenated fasta (with Uniprot) for compatibility with Fragpipe's group-specific FDR calculation is available on the following GitHub link: https://github.com/bassanilab/CircRNA_MS_ref_fasta

For manuscripts utilizing custom algorithms or software that are central to the research but not yet described in published literature, software must be made available to editors and reviewers. We strongly encourage code deposition in a community repository (e.g. GitHub). See the Nature Portfolio guidelines for submitting code & software for further information.

Data

Policy information about availability of data

All manuscripts must include a data availability statement. This statement should provide the following information, where applicable:

- Accession codes, unique identifiers, or web links for publicly available datasets
- A description of any restrictions on data availability
- For clinical datasets or third party data, please ensure that the statement adheres to our policy

MS raw files, reference fasta files and NewAnce, FragPipe and Spectronaut parameters and outputs generated in this study have been deposited in the ProteomeXchange Consortium via the PRIDE partner repository91 under accession code PXD043989 [https://proteomecentral.proteomexchange.org/cgi/GetDataset?ID=PXD043989]. The lung cancer cohort MS data used in this study are available in the ProteomeXchange Consortium under accession code PXD034772 [https://proteomecentral.proteomexchange.org/cgi/GetDataset?ID=PXD034772]. Publicly available DDA raw files derived from T1185B cells treated or not with IFN used in this study are available in the ProteomeXchange Consortium under accession code PXD013649 [https://proteomecentral.proteomexchange.org/cgi/GetDataset?ID=PXD013649]. The circBase database can be found at (http://www.circbase.org/). The UniProt database can be found at https://www.uniprot.org. The Cancer Genome Atlas (TCGA) data can be found at https://www.cancer.gov/tcga. GTEx Portal can be accessed through https://www.gtexportal.org/home/86. HLA Ligand Atlas can be accessed through https://hla-ligand-atlas.org/welcome. The Human Protein Atlas can be accessed through proteinatlas.org. Source data are provided with this paper.

Research involving human participants, their data, or biological material

Policy information about studies with human participants or human data. See also policy information about sex, gender (identity/presentation), and sexual orientation and race, ethnicity and racism.

Reporting on sex and gender	Male/female information was collected based on informed consent. This was a proof-of-concept exploratory study with a small set of samples. Sex and gender were not considered in the study design. No analysis or correlations based on sex and gender were performed.
Reporting on race, ethnicity, or other socially relevant groupings	Not applicable.
Population characteristics	Patient characteristics of samples undergoing HLA-I immunoprecipitation during this study:<br>Sample Name/Diagnosis/Gender<br>Mel-1/Melanoma/F/40y<br>T1185B/Melanoma/F/60y<br><br>Patient characteristics from the publicly available MS data from the eight lung cancer patients cohort:<br>Sample Name/Diagnosis/Gender<br>C3N-02672/LUAD/F<br>C3N-02671/LUAD/F<br>C3N-02287/LUAD/M<br>C3N-02288/LUSC/F<br>C3N-02289/LUSC/M<br>C3N-02290/LUAD/M<br>C3N-03023/LCNEC/F<br>C3N-03421/LUAD/F
Recruitment	Tissues from Mel-1 patient were collected and biobanked. This patient was selected based on sample availabitliy. Material was enough to conduct immunopeptidomics. This selection should not have any impact on the results obtained.
Ethics oversight	An informed consent was given by the participants, according to the requirements of the institutional review board (Ethics Commission, Centre hospitalier universitaire vaudois, CHUV).

Note that full information on the approval of the study protocol must also be provided in the manuscript.

Field-specific reporting

Please select the one below that is the best fit for your research. If you are not sure, read the appropriate sections before making your selection.

☒ Life sciences ☐ Behavioural & social sciences ☐ Ecological, evolutionary & environmental sciences

For a reference copy of the document with all sections, see nature.com/documents/nr-reporting-summary-flat.pdf

Life sciences study design

All studies must disclose on these points even when the disclosure is negative.

Sample size	No sample-size calculation was performed. Sample size was sufficient for this proof-of-concept study by showing that circRNA-derived

Sample size	peptides were identified in tested samples/patients.
Data exclusions	No data was excluded.
Replication	Two technical replicates were successfully performed for each of the MS measurements of DDA, DIA and PRM samples. Three biological replicates of T1185B and two biological replicates of Mel-1 were successfully used in the DDA analysis. Please see Supplementary Table 4 for detailed information on replicates for MS-based analyses. Agros gel experiments were performed once.
Randomization	Not applicable. This was a proof-of-concept exploratory study with a small set of samples.
Blinding	Not applicable. This was a proof-of-concept exploratory study with a small set of samples.

Reporting for specific materials, systems and methods

We require information from authors about some types of materials, experimental systems and methods used in many studies. Here, indicate whether each material, system or method listed is relevant to your study. If you are not sure if a list item applies to your research, read the appropriate section before selecting a response.

Materials & experimental systems

n/a	Involved in the study
☐	☒ Antibodies
☐	☒ Eukaryotic cell lines
☒	☐ Palaeontology and archaeology
☒	☐ Animals and other organisms
☒	☐ Clinical data
☒	☐ Dual use research of concern
☒	☐ Plants

Methods

n/a	Involved in the study
☒	☐ ChIP-seq
☒	☐ Flow cytometry
☒	☐ MRI-based neuroimaging

Antibodies

Antibodies used

Anti HLA-I antibody : from hybridoma “HB-95”

Company name : ATCC
Catalog number : HB-95
Lot number: 7001294
Clone name: W6/32
Antigenic determinant: HLA-A, B, C
Isotype: IgG2a
Host: mouse
Cell type: Hybridoma: B lymphocyte
Clonality: monoclonal

Validation

Validation by vendor following ATCC guidelines. Certificate of Analysis can be found here: https://www.lgcstandards-atcc.org/Products/All/HB-95.aspx?geo_country=ch#documentation

Additionally, anti-HLA-I antibody was validated directly in our laboratory, through the use of this antibody for immuno-affinity purification of HLA-I peptides from cell lines and tissue samples. These peptides were measured by mass spectrometry, and their characteristics fit that of HLA-I peptides.

Eukaryotic cell lines

Policy information about cell lines and Sex and Gender in Research

Cell line source(s)	Melanoma cell line T1185B was provided by Prof. Daniel Speiser from the Department of Fundamental Oncology, University of Lausanne. Melanoma cell line Mel-1 was provided by the Center of Experimental Therapy Biobank (CHUV), Lausanne.
Authentication	Samples were authenticated by comparing the names labelled on the vials received with the providers' information. Molecular HLA typing was performed.
Mycoplasma contamination	All cell lines tested negative for mycoplasma contamination.
Commonly misidentified lines (See ICLAC register)	No commonly misidentified cell lines were used in the study
